# Targeting ketone body metabolism in mitigating gemcitabine resistance

**DOI:** 10.1172/jci.insight.177840

**Published:** 2024-12-20

**Authors:** Krizia Rohena-Rivera, Sungyong You, Minhyung Kim, Sandrine Billet, Johanna ten Hoeve, Gabrielle Gonzales, Chengqun Huang, Ashley Heard, Keith Syson Chan, Neil A. Bhowmick

**Affiliations:** 1 Department of Medicine, Cedars-Sinai Medical Center, Los Angeles, California, USA.; 2 Samuel Oschin Cancer Center, Los Angeles, California, USA.; 3 Department of Urology and; 4 Department of Computational Biomedicine, Cedars-Sinai Medical Center, Los Angeles, California, USA.; 5 UCLA Metabolomics Center, Department of Molecular & Medical Pharmacology, UCLA, Los Angeles, California, USA.; 6 Department of Urology and Neal Cancer Center, Houston Methodist Research Institute, Houston, Texas, USA.; 7 Department of Research, VA Greater Los Angeles Healthcare System, Los Angeles, California, USA.

**Keywords:** Oncology, Cancer, Drug therapy, Urology

## Abstract

Chemotherapy is often combined with surgery for muscle invasive and nonmuscle invasive bladder cancer (BCa). However, 70% of the patients recur within 5 years. Metabolic reprogramming is an emerging hallmark in cancer chemoresistance. Here, we report a gemcitabine resistance mechanism that promotes cancer reprogramming via the metabolic enzyme OXCT1. This mitochondrial enzyme, responsible for the rate-limiting step in β-hydroxybutyrate (βHB) catabolism, was elevated in muscle invasive disease and in patients with chemoresistant BCa. Resistant orthotopic tumors presented an OXCT1-dependent rise in mitochondrial oxygen consumption rate, ATP, and nucleotide biosynthesis. In resistant BCa, knocking out *OXCT1* restored gemcitabine sensitivity, and administering the nonmetabolizable βHB enantiomer (S-βHB) only partially restored gemcitabine sensitivity. Suggesting an extrametabolic role for OXCT1, multi-omics analysis of gemcitabine sensitive and resistant cells revealed an OXCT1-dependent signature with the transcriptional repressor OVOL1 as a master regulator of epithelial differentiation. The elevation of OVOL1 target genes was associated with its cytoplasmic translocation and poor prognosis in a cohort of patients with BCa who have been treated with chemotherapy. The KO of *OXCT1* restored OVOL1 transcriptional repressive activity by its nuclear translocation. Orthotopic mouse models of BCa supported OXCT1 as a mediator of gemcitabine sensitivity through ketone metabolism and regulating cancer stem cell differentiation.

## Abstract 2

Novel gemcitabine resistance mechanism links ketones and stem-cell reprogramming via OXCT1/OVOL1 interaction.

## Introduction

Bladder cancer (BCa) is the second most common urologic malignancy with an estimated 82,290 new cases for 2023 in the United States. Male patients comprise 75% of the cases worldwide, with tobacco smoking being the primary risk factor (1). BCa can be muscle invasive BCa (MIBC) or nonmuscle invasive BCa (NMIBC), inclusive of papillary lesions, carcinoma in situ*,* and those that invade the lamina propria (2). This highly heterogeneous disease is further characterized into luminal and basal molecular subtypes with invasive and noninvasive lesions that affect clinical care decisions (3). MIBC, arising from nonpapillary or carcinoma in situ lesions, is frequently managed with neoadjuvant chemotherapy including gemcitabine-cisplatin followed by surgical intervention. However, lifestyle complications make bladder-preserving treatments a desired alternative. Metastatic recurrence is usually lethal but can be managed with chemotherapy and, more recently, with immune checkpoint inhibitors for eligible patients (4). Both genomic and metabolomic profiling provide insight into BCa progression, staging, treatment outcomes, and chemoresistance (3). Specific lipid metabolism reprogramming plays a role in BCa cisplatin resistance (5). Downstream ketone metabolites, like acetoacetate and β-hydroxybutyrate (βHB), are elevated in canine invasive BCa compared with noninvasive disease as well as in patients with MIBC compared with healthy participants (6, 7). 3-Hydroxy-3-methylglutaryl-CoA synthase 2 (*HMGCS2*) catalyzes the rate-limiting step for βHB synthesis and 3-oxoacid CoA-transferase 1 (*OXCT1*), also known as succinyl-CoA-3-oxaloacid CoA transferase 1 (*SCOT1*), catalyzes the rate-limiting step for βHB catabolism; these are key genes in ketone metabolism. Both are mitochondrial matrix enzymes associated with recurrent solid tumors of the prostate and breast among others (8, 9). While ketones can serve as an energy source for cancer cells, at early stages, βHB is considered a tumor suppressor for colon and breast cancers through niacin receptor, GPR109a, signaling (10, 11). Considering the contrasting roles of βHB, better understanding of the metabolic processes in BCa therapeutic resistance is needed.

Gemcitabine (2′,2′-difluorodeoxycytidine) is the standard of care for several cancers including pancreatic, colon, breast, ovarian, bladder, and non–small cell lung cancer (12, 13). Due to its relative lower toxicity, this DNA synthesis inhibitor can be administered as a single agent and often in combination with radiation, cisplatin, and/or taxanes (14, 15). Despite a reasonable initial response, 60%–70% of patients with BCa relapse within the first year (16). Gemcitabine resistance mechanisms have been reported to involve hedgehog, Wnt, and Notch signaling as means of cancer stem cell reprogramming, dependent on the tumor type (17). In this study, we show that elevated *OXCT1* supports gemcitabine resistance by driving anaplerosis, nucleotide biosynthesis, and *OVOL1* deregulation. Vertebrate homologs of *Drosophila* OVO (OVOL1 and OVOL2) have common and distinct transcriptional targets generally recognized to suppress stem features and epithelial-to-mesenchymal transdifferentiation (18, 19). Both *OVOL1* and *OVOL2* are zinc-finger transcription factors (TFs) that drive epithelial lineage determination (20). Accordingly, the suppression of the *OVOL1/OVOL2* signaling axis by *OXCT1* activity was found to be permissive of lineage plasticity, with broader implications for chemoresistance.

## Results

### Rate-limiting enzymes of ketone metabolism affect gemcitabine sensitivity.

**Figure 1:**
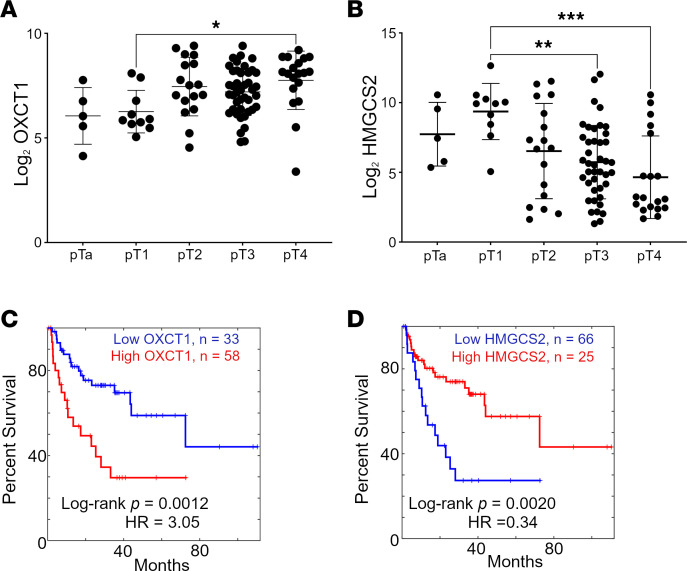
OXCT1 and HMGCS2 expression in clinical sample datasets. (**A** and **B**) High-risk BCa Riester dataset (*n* = 93) was used to identify OXCT1 (**A**) and HMGCS2 (**B**) expression by disease stage (23). Statistical analysis done with 1-way ANOVA at 95 % CI. *P < 0.05, **P < 0.01, ***P < 0.001. (**C**) The Peter C. Black chemotherapy treated cohort (*n* = 91) was used to identify OXCT1 expression correlates with poor outcome within (*P* = 0.0012). (**D**) HMGCS2 expression correlates with survival after chemotherapy (*P* = 0.0020) (24).

Metabolic reprogramming is a hallmark of cancer due in part to the high energetic demands required to sustain tumor expansion. Specifically, ketone metabolites like acetoacetate and βHB are associated with increased staging (21). With the clinical significance of ketone metabolites in mind, we explored the expression of crucial genes for ketogenesis (*HMGCS2*) and ketolysis (*OXCT1*). A Kaplan-Meier plot demonstrated that elevated *OXCT1* expression was significantly associated with poor survival in the TCGA urothelial BCa dataset (*n* = 408), and the Mattias Höglund BCa dataset (*n* = 308; Supplemental Figure 1, A and C; supplemental material available online with this article; https://doi.org/10.1172/jci.insight.177840DS1) (22). Conversely, *HMGCS2* expression was positively associated with survival in the same datasets (Supplemental Figure 1, B and D). In addition, the Markus Riester high-risk cohort BCa dataset (*n* = 93) revealed that *OXCT1* was significantly expressed in invasive disease (pT4) while *HMGCS2* was downregulated at higher disease stages, showing a correlation with muscle invasive disease (Figure 1, A and B) (23). To assess a relationship with chemoresistance, we focused on patients with MIBC who underwent neoadjuvant chemotherapy in the Peter C. Black dataset (*n* = 91), which showed that *OXCT1* and *HMGCS2* have opposing correlations with survival (Figure 1, C and D) (24). Altogether, *OXCT1* and *HMGCS2* consistently relate to the overall survival, staging, and, importantly, the outcome of chemotherapy treatment of patients with BCa.

**Figure 2:**
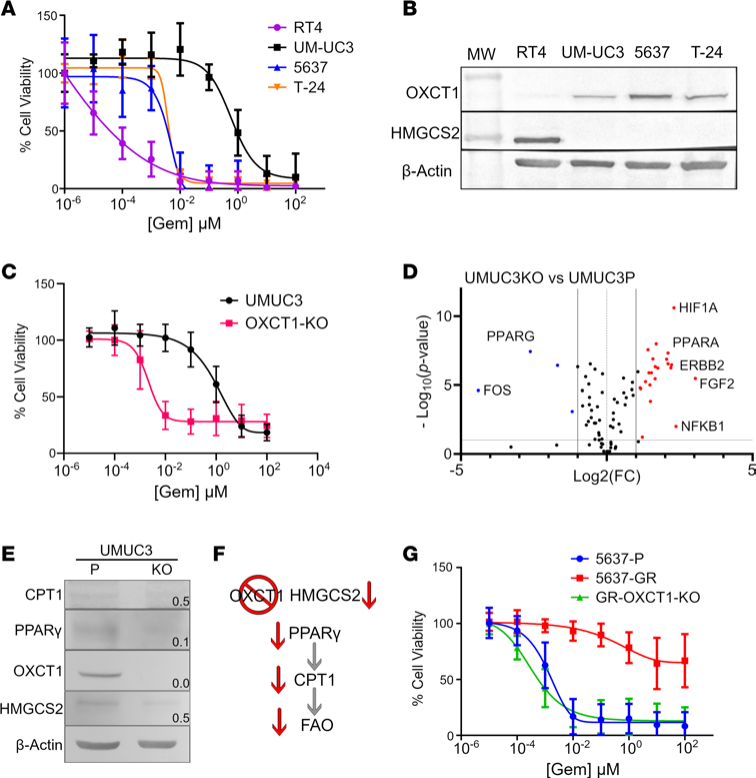
OXCT1 Expression affects gemcitabine response. (**A**) BCa cell line panel and their response to gemcitabine. All cell lines were treated with gemcitabine in various logarithmic concentrations to determine their IC_50_ at 72 hours using an MTT assay. (**B**) OXCT1 and HMGCS2 expression was measured by Western blot in naive cells after 72 hours of culture. (**C**) Knocking out OXCT1 in UM-UC3 cell lines causes a significant shift in gemcitabine IC_50_. (**D**) Drug resistance PCR array shows significant changes in PPAR signaling pathway. (**E**) Western blot of parental UM-UC3 (P) and isogenic OXCT1KO (KO) have decreased expression of HMGCS2, PPAR**γ**, and downstream CPT1. Calculations shown are normalized to β-actin and relative to the parental line. (**F**) PCR array and Western blot results suggest a metabolic shift that could decrease FAO in OXCT1-KO cells. (**G**) Cell viability of the isogenic 5637 cells were measured following incubation with gemcitabine at indicated concentrations.

DNA synthesis inhibitors like gemcitabine serve as a tool to examine the crosstalk between gene regulation and metabolism. Initially, we evaluated the expression of enzymes responsible for ketone body metabolism in a diverse panel of BCa cell lines and their effect on gemcitabine sensitivity. A transitional papillary line, RT4, was the most sensitive to gemcitabine with an IC_50_ of < 0.1 nM. A transitional carcinoma line, UM-UC3, was the most resistant with an IC_50_ of 600 nM, whereas the 5637 cell line (muscle invasive) and T24 (transitional carcinoma) lines had a very similar gemcitabine sensitivity with IC_50_ of 4 nM (Figure 2A). In this cell line panel, only RT4 cells had detectable HMGCS2 expression, while UM-UC3, the 5637 cell line, and T24 only expressed OXCT1 by Western blotting (Figure 2B). The cell line data support HMGCS2 expression in less aggressive gemcitabine-sensitive BCa and suggested that OXCT1 may play a role in more aggressive disease and gemcitabine resistance. Since the UM-UC3 had elevated OXCT1 expression and showed an intrinsic resistance to gemcitabine, we used CRISPR/CAS9 to generate a stable KO of *OXCT1* (UM-UC3-OXCT1KO) cell line (Supplemental Figure 2A). The loss of *OXCT1* expression induced gemcitabine sensitivity with a significantly lower IC_50_ of 2 nM (Figure 2C). Parental UM-UC3 and UM-UC3-OXCT1KO gene expression specific to drug resistance pathways was investigated with a PCR array. Data show differences in peroxisome proliferator activated receptor γ (*PPARG*); peroxisome proliferator activated receptor α (*PPARA*); fos-proto-oncogene, AP-1 TF subunit (*FOS*); hypoxia-inducible factor 1 subunit α (*HIF1A*); Erb-B2 receptor tyrosine kinase 2 (*ERBB2*); fibroblast growth factor 2 (*FGF2*); andNF-κB subunit 1 (*NFKB1*) (Figure 2D). These results suggest a possible metabolic shift in the UM-UC3-OXCT1KO. *HIF1A* is a metabolic regulator known to reduce oxidative processes and to inhibit *PPARG* in favor of an anaerobic metabolic shift (25). Using Western blotting, we confirmed that OXCT1 KO resulted in a decrease in *PPARG* expression as well as downstream *CPT1*, responsible for fatty acid entry into the mitochondria (26). Furthermore, *HMGCS2* expression remained low in the UM-UC3-OXCT1KO cell line, suggesting that both ketogenesis and ketolysis were inhibited (Figure 2, E and F). Among other genes significantly altered by the *OXCT1* KO, a member of the epidermal growth factor receptor (*EGFR*) family, *ERBB2*, was downregulated. This oncogene is known to be amplified in BCa. Interestingly, gemcitabine treatment is reported to elevate *ERBB2* expression, making it a candidate target in breast, pancreatic, and biliary tract cancer treatment (27). The alterations in *OXCT1* expression in the UM-UC3 line suggest its role in gemcitabine sensitivity.

Next, we developed an acquired resistance model with the 5637 cell line. The stable gemcitabine-resistant cell line (5637GR) was created through continuous gemcitabine exposure to achieve an IC_50_ > 100 μM. Expression analysis showed that 5637GR had significantly elevated *OXCT1* expression when compared its isogenic parental line (*P* = 0.033; Supplemental Figure 2B). The subsequent KO of *OXCT1* of the 5637GR cells using CRISPR/Cas9 (GR-OXCT1KO) resulted in a dramatic restoration of gemcitabine sensitivity, near to that of the 5637P line (5637P; IC_50_ = 0.001 μM; Figure 2G). Together, our data support a robust relationship between *OXCT1* expression and gemcitabine resistance in both acquired and intrinsic models.

### Metabolic changes induced by OXCT1 orchestrate gemcitabine response.

**Figure 3:**
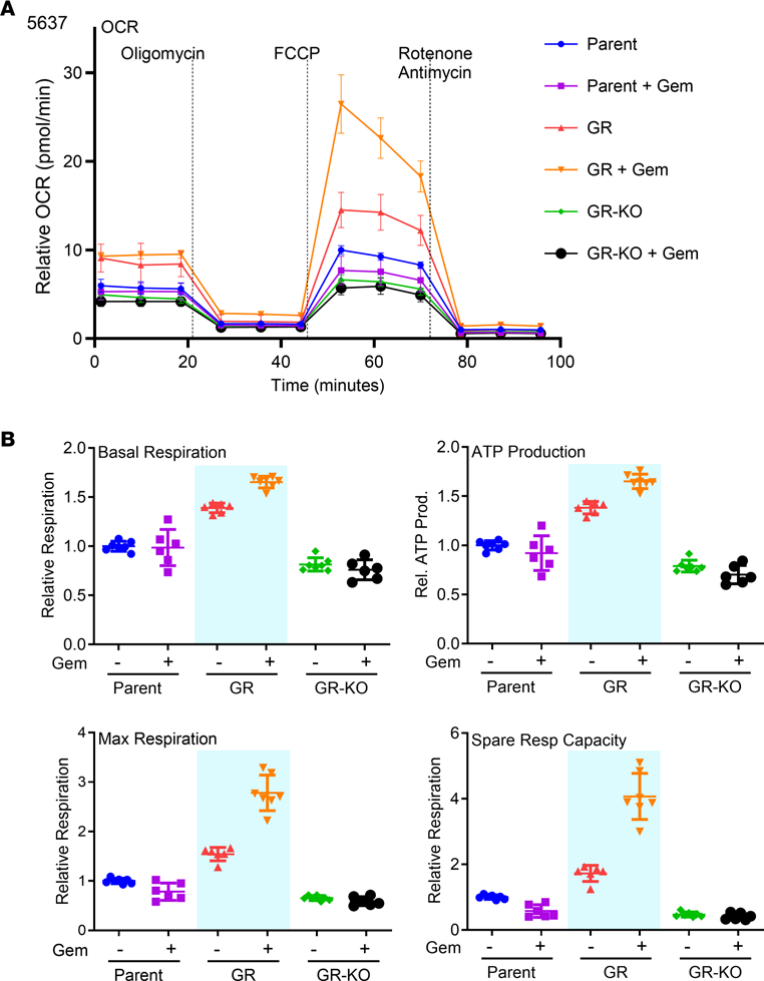
Gemcitabine resistance is affected by cell respiration. (**A**) Oxygen consumption rate (OCR) under acute gemcitabine was measured using the Seahorse XFe24. (**B**) Isogenic 5637 lines, parental, 5637GR (GR), and 5637GR-KO (GR-KO) cells had differential metabolic activity. Acute addition of gemcitabine affected basal respiration, ATP production, maximal respiration, and spare respiratory capacity.

Based on the role of OXCT1 in ketone catabolism, we examined the differential metabolic effect in the 5637 cell line acquired resistance model. For these studies, Seahorse XF measurements of oxygen consumption rate (OCR) were performed while serially manipulating the different mitochondrial respiratory chain complexes, as previously described (28). Basal, maximal, and spare respiratory capacity of 5637GR was significantly greater than that of the 5637P or the GR-OXCT1KO lines (Figure 3A). Interestingly, acute gemcitabine (100 nM) treatment caused a significant increase in basal, maximal, and spare respiratory capacity in the 5637GR cells when compared with vehicle, not observed in the 5637P or GR-OXCT1KO cells (Figure 3B). Calculated ATP-linked respiration was also greater in 5637GR than either parental or GR-OXCT1KO lines. This was further enhanced by gemcitabine treatment. The GR-OXCT1KO cells had a significantly lower spare respiratory capacity (the difference between basal ATP production and its maximal activity) compared with the parental line. Moreover, basal respiration and maximal respiration remained unchanged by the addition of gemcitabine, and spare respiratory capacity remained low. Thus, *OXCT1* KO caused a metabolic shift toward anaerobic metabolism, yielding lower ATP production; this suggests that *OXCT1* expression is necessary for an elevated respiration and survival under gemcitabine treatment.

**Figure 4:**
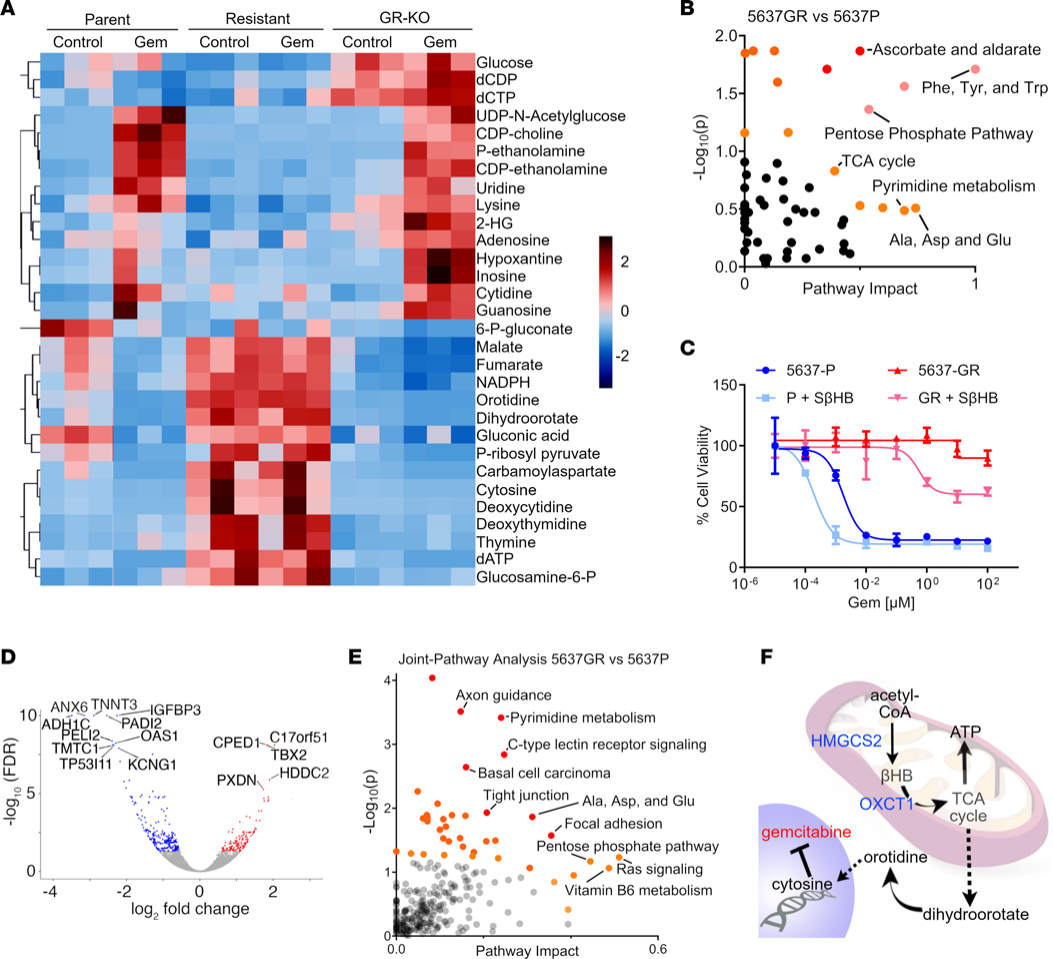
Metabolomic analysis of isogenic bladder cancer lines revealed role of βHB in gemcitabine sensitivity. (**A**) Heatmap of the top 30 metabolites relative to the untreated 5637P cells. Gemcitabine-resistant cells have an increase in nucleotide synthesis regardless of treatment. OXCT1-KO cells show a decrease in TCA cycle intermediates while accumulating glucose, suggesting a metabolic shift. (**B**) Metabolomic pathway analysis between parental and gemcitabine-resistant cells. (**C**) Cell viability assay was performed on the indicated cell lines with the metabolically inactive βHB enantiomer (S-βHB). (**D**) Volcano Plot showing all the top upregulated and downregulated genes in acquired resistance. (**E**) Multi-omics joint-pathway analysis showing the crosstalk between metabolomics and gene expression; significantly altered pathways between parental and gemcitabine resistance are highlighted. (**F**) Graphic depiction of the utilization of βHB in the mitochondria. Metabolites from the TCA cycle can be used for nucleotide synthesis.

Next, the metabolic changes induced by 72 hours of gemcitabine treatment were evaluated using mass spectrometry (MS). Overall, 147 metabolites were identified by targeted analysis, of which 77 demonstrated significant differential enrichment by 1-way ANOVA post hoc analysis. Results highlight metabolites involved in pyrimidine synthesis (orotidine, dihydroorotate, and cytidine), TCA cycle (malate, fumarate, and succinate), and the pentose phosphate pathway (ribose-5-phosphate, gluconic acid, and sedoheptulose-7-phosphate; Supplemental Table 1). A heatmap of hierarchical clustering of the top 30 differentially enriched metabolites was generated relative to the untreated 5637P cells (Figure 4A). Notably, glucose was enriched in GR-OXCT1KO upon gemcitabine treatment but not in 5637GR cells. This result shows that gemcitabine treatment itself affected metabolism, and it also confirms that GR-OXCT1KO cells have a unique metabolism compared with resistant cells. On the other hand, cytosine, orotidine, NADPH, and phosphoribosyl diphosphate (PRPP), involved in nucleotide biosynthesis, were uniquely elevated in 5637GR cells regardless of gemcitabine treatment. The GR-OXCT1KO cells restored these same metabolites to parental levels, suggesting that *OXCT1* expression has an effect on nucleotide biosynthesis pathways. Pathway enrichment analysis between untreated parental and 5637GR showed pyrimidine metabolism as among one of the significantly altered pathways in the development of gemcitabine resistance (Figure 4B and Supplemental Table 2). Amino acid metabolism (phenylalanine, cysteine, and alanine) as well as the ascorbate and aldarate metabolism were differentially regulated in 5637GR when compared with the 5637P cells (Figure 4B). The latter is known for being a source of oxidative stress protection, also suggesting that resistant cells have an increase in aerobic metabolism (29). The known functions of OXCT1 involving the TCA cycle as well as CoA synthesis were also identified (Supplemental Table 2). Upon gemcitabine treatment, these pathways were more significantly enriched in the resistant cells, including ketone body metabolism (Supplemental Figure 3A and Supplemental Table 3). To identify which metabolic signature precedes gemcitabine resistance, the gemcitabine-sensitive isogenic lines GR-OXCT1KO and 5637P cells were compared after 72 hours of gemcitabine treatment. Our results establish that knocking out OXCT1 caused an accumulation of palmitate and reduced carnitine, suggesting that fatty acid oxidation (FAO) is decreased in favor of anabolic processes (Supplemental Figure 3B). Interestingly, PRPP was significantly decreased in GR-OXCT1KO, highlighting the increase observed in 5637GR. Thus, the enhancement in nucleotide biosynthesis associated with gemcitabine resistance seems to be *OXCT1* expression dependent. Furthermore, the metabolic pathway enrichment analysis between the sensitive lines showed that oxidative processes were inhibited in GR-OXCT1KO compared with the parental cell line. In contrast, fatty acid biosynthesis was enriched in GR-OXCT1KO cells (Supplemental Figure 3C).

Elevated expression of *OXCT1* in gemcitabine-resistant cells can deplete βHB, while targeting *OXCT1* would cause its accumulation. Therefore, to simulate the metabolic effect of targeting *OXCT1*, we exploited the chiral property of βHB by treating the cells with the metabolically inactive enantiomer S-βHB (5 mM) at a dose range of natural ketosis without causing severe ketoacidosis (30, 31). Chiral molecules have nonsuperimposable mirror structures, where in this case, R-βHB is metabolically active and S-βHB is not. Treating the 5637P cells with S-βHB for 72 hours supported gemcitabine sensitivity with a significant reduction in IC_50_ (*P <* 0.001; Figure 4C). While a partial restoration of gemcitabine sensitivity was observed when the 5637GR cells were treated with S-βHB (*P <* 0.001), the extent of sensitization did not approach that of the parental line. This result reinforces that OXCT1 may have a role outside of metabolism in support gemcitabine sensitivity.

To further investigate the role of OXCT1 in gemcitabine resistance, we used an integrated multi-omics approach. Gene expression changes were evaluated in 3 independent clones of 5637P and 5637GR cells. These independently derived single-cell clones consistently demonstrated elevated *OXCT1* expression in the 5637GR lines (Supplemental Figure 4A). Upregulated genes included T-box TF 2 (*TBX2*), cadherin like and PC-esterase domain containing 1 (*CPED1*), and peroxidasin (*PXDN*) associated with poor outcomes (32–34). The downregulated genes included annexin A6 (*ANXA6*, involved in autophagy), troponin T3 (*TNNT3*, involved in skeletal muscle development), and peptidyl arginine deiminase, type II (*PADI2*, involved in protein citrullination) (Figure 4D) (35–37). The top 10 differentially regulated genes were validated by quantitative PCR (qPCR) (Supplemental Table 4). Afterward, all the differentially expressed genes (Supplemental Table 5) and all the differentially enriched metabolites of 5637GR and 5637P (Supplemental Table 2) were integrated in a joint-pathway multi-omic analysis. Here, we equally considered the statistical contribution of metabolomics and transcriptomics to enrich metabolic and signaling pathways (38). Our results indicate gemcitabine resistance to be associated with pyrimidine metabolism and the pentose phosphate pathway, while also highlighting axon guidance and focal adhesion (Figure 4E). These findings suggest gemcitabine resistance involved OXCT1-mediated changes in metabolism and cell differentiation. Here, gemcitabine-induced OXCT1 expression mediated ATP generation through fatty acid oxidation (FAO) energy required for dihydroorotate and UMP synthesis via orotidine monophosphate (Figure 4F). One mechanism for gemcitabine resistance could involve the generation of cytosine to help overcome gemcitabine integration into the newly synthesized DNA in a competitive manner (39, 40).

### OXCT1 serves as a master regulator for stem cell reprogramming.

**Figure 5:**
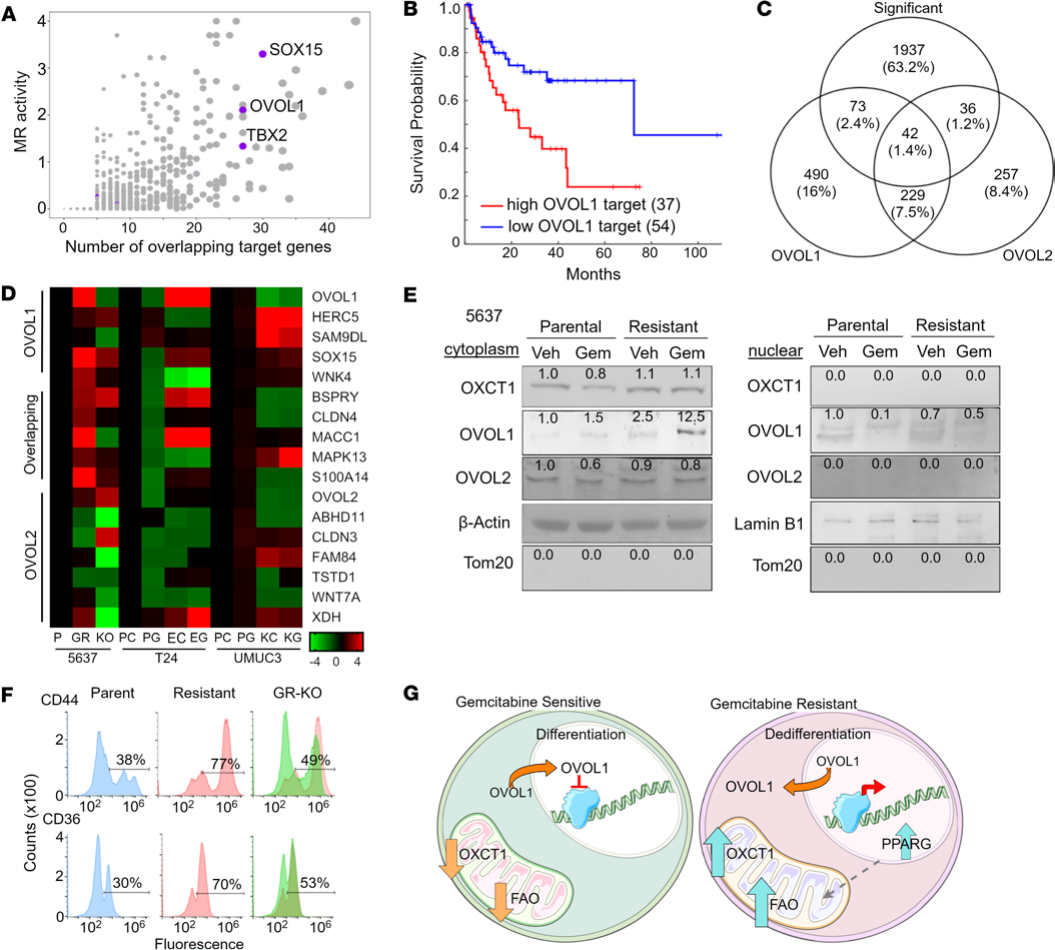
Genomic analysis of isogenic bladder cancer lines revealed OVOL1 as a master regulator. (**A**) Master regulator analysis highlighting the significant transcription factors *SOX15*, *OVOL1*, and *TBX2*. (**B**) Survival curve associating *OVOL1* targets in pretreatment and gemcitabine/cisplatin-treated samples from the Peter C. Black dataset (HR = 2.35, *P* = 0.0119) (24). (**C**) Venn diagram highlighting the proportion of *OVOL1* and OVOL2 targets in the differentially expressed genes. More details in Supplemental Tables 5 and 6. (**D**) qPCR confirmation of the top OVOL1 and OVOL2 targets — 5637: P (parental), GR (gemcitabine resistant), KO (GR-OXCT1 KO); T24: PC (parental, untreated), PG (parental, gemcitabine-treated), EC (gemcitabine-exposed, untreated), EG (gemcitabine-exposed, gemcitabine treated); and UM-UC3: PC (parental, untreated) PG (parental, gemcitabine-treated) KC (OXCT1-KO, untreated), KG (OXCT1-KO, gemcitabine treated) — all normalized to their respective control. Resistant or exposed cells show a higher expression of OVOL1 targets suggesting that OVOL1 is not repressing its targets. Heatmap represents fold change to each respective control. (**E**) Representative Western blot images for OXCT1, OVOL1, and OVOL2 using β-actin, lamin B1, and Tom20 as cytoplasmic, nuclear, and mitochondrial markers, respectively. Representative densitometry values were normalized to their respective loading control and relative to the parental vehicle sample. Western blots were repeated 4 times. (**F**) Cell surface CD44 and CD36 were measured by flow cytometry of indicated lines following 72 hours gemcitabine treatment at IC_50_ doses. (**G**) Schematic representation of the roles of OXCT1 and OVOL1 in gemcitabine sensitive and gemcitabine resistant cells. Under lower OXCT1, OVOL1 is free to translocate to the nucleus and repress target genes including *PPARG*, promoting a more differentiated state and reduced FAO. Under gemcitabine-resistant conditions, there is higher OXCT1, and cytoplasmic OVOL1 promotes PPARG, FAO, and a more dedifferentiated state.

Master regulator analysis of the RNA-Seq results helped us identify TFs orchestrating differentially expressed target genes (41, 42). Master regulator analysis revealed 3 key TFs, *TBX2*, *SOX15*, and *OVOL1*, in support of gemcitabine resistance (Figure 5A). These transcription regulators and their corresponding downstream targets were evaluated in the Peter C. Black dataset that included a gemcitabine/cisplatin-treated cohort (24). There was a strong correlation with the expression levels of OVOL1 downstream targets (more than 800 targets) and poor prognosis, not observed for *TBX2* or *SOX15* (Figure 5B; Supplemental Figure 4, B and C; and Supplemental Table 6). Of note, OVOL2 downstream targets, some overlapping with OVOL1, were also identified. Interestingly, *PPARG* is among the unique *OVOL1* targets. Within the differentially expressed genes in our dataset, 150 were OVOL1/OVOL2 pathway targets, with 73 being unique to *OVOL1* and 36 unique to *OVOL2* (*P* < 0.05; Figure 5C). These data suggest that *OVOL1* and *OVOL2* regulation of differentiation might be involved in gemcitabine resistance (43). Both regulators are known as transcriptional repressors of EMT in favor of epithelial differentiation (20, 44). Importantly, the indicated overexpression of their targets would suggest reduced repressor activity. To test this assumption, the top *OVOL1* and *OVOL2* downstream targets were measured in 5637P, 5637GR, and 5637GR-OXCT1KO lines by qPCR (Figure 5D). Notably, several targets upregulated in 5637GR were found to be restored to near parental levels when *OXCT1* was knocked out (GR-OXCT1KO lines), supporting a regulatory relationship between *OXCT1* expression and *OVOL1* activity. These findings were further tested in UM-UC3 and T24 bladder lines. As with the isogenic 5637 lines, the KO of *OXCT1* in the inherently resistant UM-UC3 cells (UM-UC3-OXCT1KO) demonstrated elevated *OVOL1* target gene expression when compared with its parental line. In contrast to the UM-UC3 line, T24 cells were exposed to IC_50_ concentrations of gemcitabine for a minimum of 4 passages. This generated a cell line with resistance to gemcitabine but more sensitive than 5637GR (T24E; Supplemental Figure 5A). These cells demonstrated elevated *OXCT1* upon gemcitabine treatment (Supplemental Figure 5B). Accordingly, *OVOL1* and *OVOL1/OVOL2* targets were upregulated in the exposed cell line; however, unique OVOL2 target genes seemed consistently downregulated even when exposed to gemcitabine (Figure 5D). Together, the multiple examples led us to reason that *OXCT1* expression regulates *OVOL1* to mediate gemcitabine resistance. To test this hypothesis, we evaluated *OVOL1* subcellular localization in the parental and resistant cell lines. We found that *OVOL1* cytoplasmic localization was greatest in the resistant isogenic lines including 5637GR, UM-UC3 parental, and T24 exposed (Figure 5E and Supplemental Figure 5, C and D). Of note, *OVOL2* remained localized to the cytoplasm. These findings suggest that gemcitabine treatment reduced nuclear availability of *OVOL1* through the upregulation of *OXCT1*.

As *OVOL1* plays a pivotal role in regulating epithelial differentiation (45), we reasoned that the loss of such signals would result in dedifferentiation and, potentially, the expression of stem features often associated with therapy resistance. Therefore, cell surface stem markers CD44 and CD36 were evaluated in cells exposed to 10 nM gemcitabine for 72 hours using FACS. The 5637GR cells had double the CD44 positivity (77%) compared with its isogenic parental line (38%; Figure 5F). The deletion of OXCT1 in the gemcitabine-resistant line reverted CD44 expression to near parental line levels (49%). Similarly, CD36 expression was significantly higher in 5637GR cells (70%) compared with the 5637P cells (30%), and the GR-OXCT1KO cells had reduced CD36 expression (53%). Subsequent hanging drop sphere-forming assays performed with the isogenic lines supported the 5637GR cells generating large spheres as having stem features compared with the parental and GR-OXCT1KO cells under control conditions (Supplemental Figure 6A). Only 5637GR cells were able to maintain sphere integrity in the presence of gemcitabine. The elevated expression of the additional stem marker, SOX9, and migration by 5637GR cells was found to be a OXCT1-dependent response, as both were limited in the GR-OXCT1KO cells, like the parental line (Supplemental Figure 6, B and C). These data support gemcitabine-induced OXCT1 as a mediator of dedifferentiation through negative regulation of OVOL1 activity (Figure 5G).

### OXCT1 affects tumor growth and gemcitabine sensitivity in mouse models.

**Figure 6:**
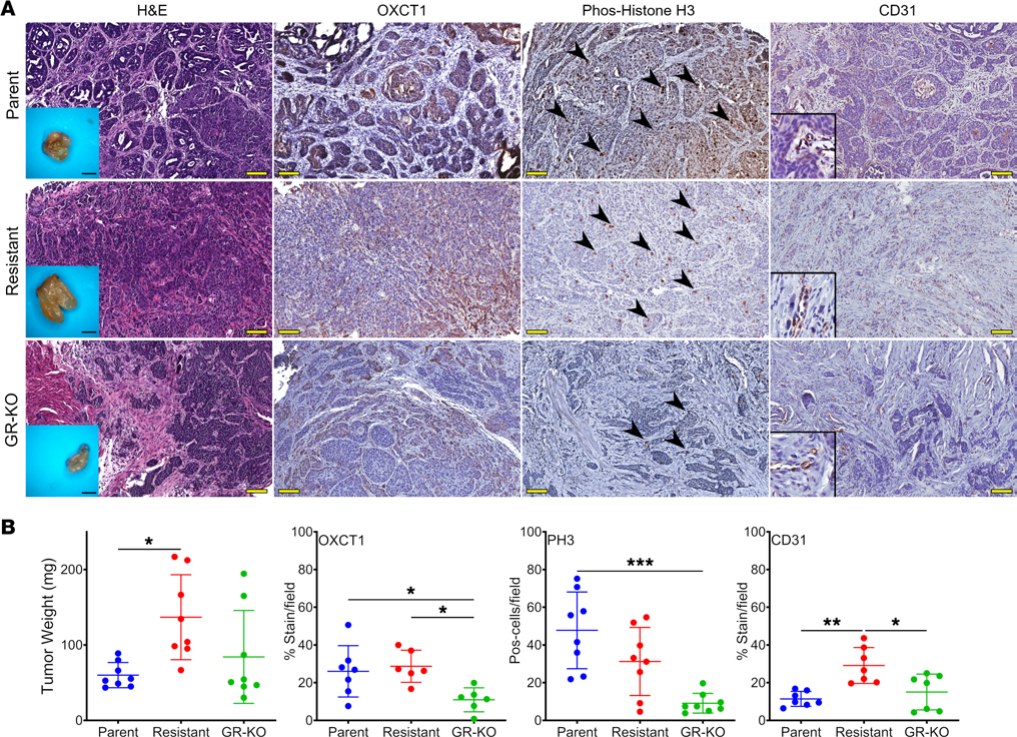
OXCT1 expression in bladder orthotopic models. (**A**) Isogenic parental (5637P), gemcitabine resistant (5637GR), and resistant OXCT1-KO (GR-KO) were injected intramurally and allowed to grow. Inset scale bar: 5 mm. The tumor histology and immunostaining were performed to evaluate OXCT1 expression, mitotic capacity (phospho-Histone H3), and vascularity (CD31). Total original magnification, 100×. Scale bar: 100 μm. Zoom: 100 μm × 100 μm. (**B**) Statistical analysis of the staining quantification was done with 1-way ANOVA at 95 % CI. *P < 0.05, **P < 0.01, ***P < 0.001 (*n* = 8). Arrowheads point to positively stained nuclei.

**Figure 7:**
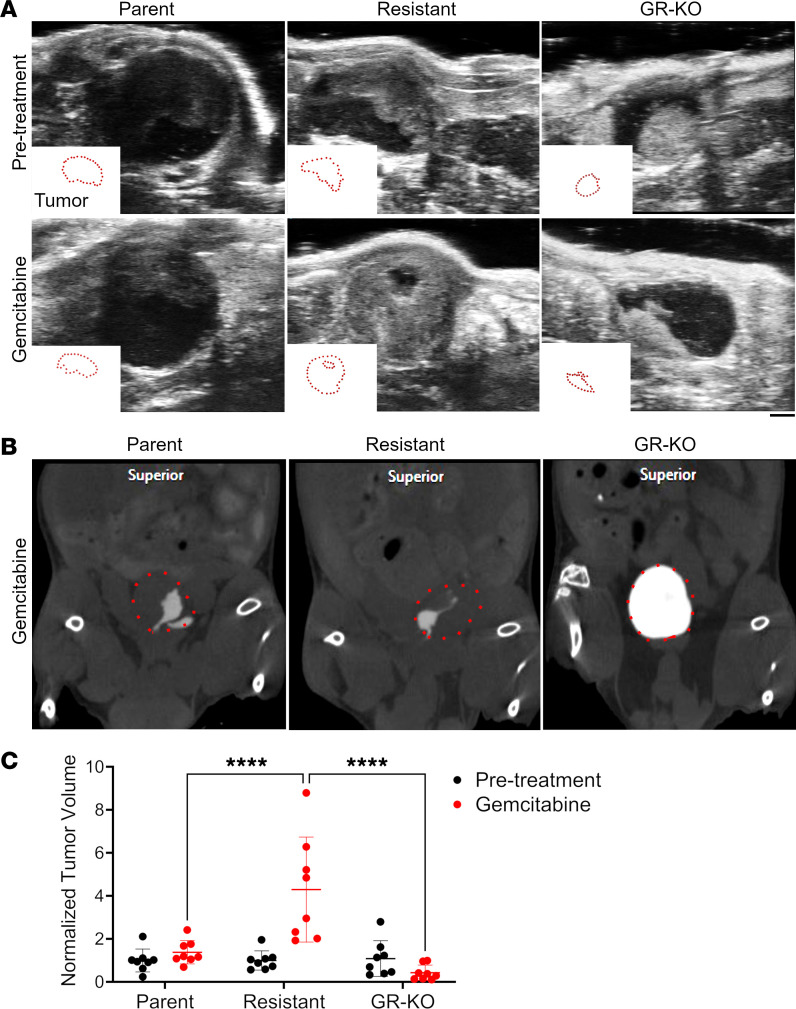
Imaging of gemcitabine treatment of orthotopic bladder cancer models. (**A**) Representative ultrasound images of 5637P, gemcitabine-resistant, and GR-OXCT1KO orthotopic bladder tumors before and after gemcitabine treatment. An outline of the tumor area is depicted in each panel. Scale bar: 1.6 mm. (**B**) Representative μCT images with iopamidol contrast after gemcitabine treatment. Quantum GX2 microCT (Perkin Elmer) X-Ray kV: 90kV, X-Ray µA 88 µA, FOV: 72 mm, Pixel size: 144 µm. (**C**) Normalized quantification of tumor volume before and after treatment as measured with ultrasound, 2-way ANOVA at 95 % CI. ****P < 0.0001 (*n* = 8 per group).

**Figure 8:**
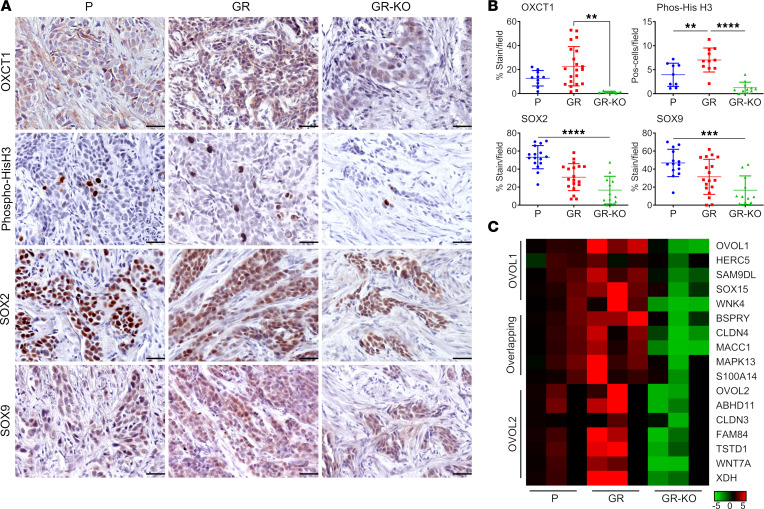
OVOL1 and stem features downstream of OXCT1 determine bladder cancer gemcitabine sensitivity. (**A**) Representative staining images of OXCT1, phospho-Histone H3, CD31, SOX2, and SOX9 in tumor tissue sections. Total original magnification, 200×. Scale bar: 50 μm. (**B**) Positive staining quantification. Statistical comparison made with 1-way ANOVA at 95 % CI. **P < 0.01, ***P < 0.001, ****P < 0.0001 (*n* = 8). (**C**) qPCR confirmation of downstream OVOL1, overlapping and OVOL2 targets in bladder tumor samples are depicted in a heatmap. Fold change scale is relative to the mean of the parental samples per row.

To determine whether OXCT1 expression would affect tumor growth, the isogenic lines were orthotopically grafted in NOD SCID-γ mice. By 8 weeks, the 5637GR tumors were significantly larger than the parental counterparts (*P* < 0.0001; Figure 6, A and B). Knocking out *OXCT1* in the 5637GR line produced tumors more variable in size than the parental line. Immunostaining was performed to localize OXCT1 expression. The tumor mitotic index and vascularity was determined by localizing phosphorylated histone H3 and CD31, respectively. Knocking out *OXCT1* significantly diminished the mitotic index compared with 5637P (*P <* 0.001; Figure 6B). The greater tumor weight of the resistant line could be attributable to the significant increase in vascular infiltration, as confirmed by the elevation in CD31 expression (*P <* 0.05). The GR-OXCT1KO and parental tumors were similar in terms of associated vasculature. Finally, we tested if targeting *OXCT1* would sensitize tumors to gemcitabine. Orthotopic grafts of 5637P, 5637GR, and GR-OXCT1KO lines were monitored for tumor growth by ultrasound and μCT imaging (Figure 7, A and B). Ultrasound measurements were found to be comparable with the final gross measurement of the extracted tumors (Supplemental Figure 7). Tumor volume measurements calculated from ultrasound imaging suggested that 5637GR tumors thrived under gemcitabine treatment while the parental and GR-OXCT1KO tumors had limited growth (Figure 7C). The 5637GR tumors were confirmed to have greater OXCT1 expression compared with the parental tumors, with negligible detection in the GR-OXCT1KO tumors by IHC (Figure 8, A and B). The parental and GR-OXCT1KO tumors had significantly fewer mitotic cells compared with the 5637GR tumors in response to gemcitabine (*P <* 0.0001). We observed no significant differences in CD31^+^ vasculature and TUNEL positivity in response to gemcitabine among the isogenic lines (Supplemental Figure 8). However, GR-OXCT1KO tumors had significantly lower stem-associated SOX2 and SOX9 expression compared with either 5637P or 5637GR tumors (*P* < 0.001). Although available antibodies did not allow for OVOL1 localization in the tissues, *OVOL1*, *OVOL2*, and their respective downstream targets, inclusive of those associated with stem features, were reliably upregulated in the 5637GR tumors compared with the parental and GR-OXCT1KO counterparts by qPCR (Figure 8C). Together, OXCT1 was identified to orchestrate metabolic and transcriptomic changes downstream of OVOL1 that contributed to the development of gemcitabine resistance.

## Discussion

In this study, we report that *OXCT1* expression is important for intrinsic and acquired gemcitabine resistance via a metabolic and genetic crosstalk. The balance between ketogenesis (catalyzed by HMGCS2) and ketolysis (catalyzed by OXCT1) is relevant to the abundance of βHB as a metabolite and signaling molecule. We found that OXCT1 expression negatively correlated with survival of patients with BCa, both in terms of tumor stage and response to therapeutic intervention (Figure 1 and Supplemental Figure 1). A recent study evaluating genomics in a MIBC cohort reported that, while the mutational burden did not increase with chemotherapy, treatment-induced clonal heterogeneity was significantly amplified (46). A diverse BCa cell panel helped support the correlative findings in patients whose *HMGCS2* loss and *OXCT1* gain were consequential to establishing gemcitabine resistance. The development of a gemcitabine-resistant cell line with chronic exposure to gemcitabine resulted in *OXCT1* enrichment. On the other hand, knocking out *OXCT1* induced gemcitabine sensitivity in an intrinsically resistant cell line (UM-UC3) as well as in the acquired resistant model (5637GR; Figure 2). Furthermore, a PCR array focused on drug resistance pathways highlighted the downregulation of *PPARG* as a result of OXCT1 loss, suggesting oxidative metabolism as a component of gemcitabine resistance. Looking at the OCR, gemcitabine-resistant cells had greater energetic capacity and ATP production. This was attributed to the elevated OXCT1 expression, as the *OXCT1* KO caused lower energetic capacity (Figure 3).

Metabolic reprogramming is a recognized mechanism for tumor growth and has been associated with chemoresistance. A hallmark of cancer stem cells is adaptation to starvation-induced nutritional stress by the generation of βHB and acetoacetate (47, 48). βHB utilization can be achieved through altered biosynthetic or catabolic mechanism, ultimately having a favorable HMGCS2/OXCT1 ratio. We found that elevated *OXCT1* in aggressive BCa parallels levels in patients with colon cancer and *HMGCS2* loss promoting tumor progression (49). Correspondingly, elevated βHB generated by the gut microbiome can reduce incidence of colon cancer by limiting inflammation and histone deacetylase activity (50). However, in the context of chemotherapy (i.e., oxaliplatin), elevated *HMGCS2* expression in the epithelia is associated with colon cancer recurrence (51). The apparent divergent roles of ketone metabolism and HMGCS2/OXCT1 expression ratio prompted us to understand chemotherapy response and resistance mechanisms. When gemcitabine-sensitive cells (parental or GR-OXCT1KO ) were acutely exposed to gemcitabine, they showed a differential metabolic signature distinct from the resistant lines. Particularly, the GR-OXCT1KO cells did not have an elevation of carnitine, suggesting that β oxidation ceased to be a viable metabolic pathway for survival (Figure 4, Supplemental Tables 2, and Supplemental Table 3). An integrated pathway analysis combining RNA-Seq and metabolomics showed a robust nucleotide biosynthesis signature in the 5637 GR cells. RNA-Seq data pointed to the transcriptional repressor *OVOL1* as a master regulator of gemcitabine resistance. Others have demonstrated the critical role *OVOL1/OVOL2* signaling has on mammary, testis, kidney, and skin epithelial development (18). We must note that our multi-omics approach also highlighted pathways associated with axon guidance and focal adhesion, which are also associated with OVOL1 activity (52). The role of *OVOL1* in skin tissue turnover and self-renewal is associated with observed lineage plasticity in the gemcitabine-resistant cells with elevated OXCT1 (53). Importantly, the OVOL1 repressive activity supported epithelial differentiation. Paradoxically, *OVOL1* expression was associated with poor overall survival and gemcitabine resistance in multiple cell lines. The identification of cytoplasmic OVOL1 localization meant that its transcriptional repression activity could not be fulfilled, despite its transcriptional elevation (Figure 5). Elevated *OVOL1* expression has been recognized in ovarian, breast, lung, and gastric cancers compared with their benign counterparts (52). The localization of OVOL1 in cytoplasmic and nuclear fractions showed that, in resistant cells, OVOL1 is elevated in the cytoplasmic fraction but depleted from the nucleus in a OXCT1-dependent manner. Whereby the KO of *OXCT1* resulted in OVOL1 translocation to the nucleus, repressing its targets to support epithelial differentiation (Figure 5 and Supplemental Figure 4). Metabolically, among the OVOL1 targets, *PPARG* mediates FAO. Although we found no evidence of a physical interaction among OXCT1, OVOL1, and PPARG, 1 direct target of OVOL1 is *MAFF* (Supplemental Table 5). In a study published by Wang et al., OVOL1-MAFF interaction was found to contribute to reactive oxygen species regulation in an extended network with several peroxiredoxins, C-MYC, MAPK13, FOS, and other enzymes involved in cholesterol biosynthesis (54). Furthermore, Ravasi et al. in a high throughput protein interaction study reported MAFF-PPARG interaction, to support OVOL1 regulation of cell differentiation and metabolism (55). We demonstrated the stem marker and fatty acid receptor CD36 to be upregulated by OXCT1. Accordingly, one can reason that OXCT1 downregulation would cause a metabolic shift toward fatty acid biosynthesis.

By focusing on *OXCT1* expression in gemcitabine-sensitive cell lines, we were able to identify OXCT1-associated mechanisms that precede gemcitabine resistance. In summary, this metabolic signature promotes anaplerosis and nucleotide biosynthesis under gemcitabine treatment as observed by the upregulation of PRPP, ATP, and orotate. Given that gemcitabine can mimic deoxycytidine, de novo pyrimidine synthesis becomes a significant pathway to eliminate the drug. However, if ATP production cannot sustain a continuous demand of nucleotides to inhibit gemcitabine integration in a competitive manner, the cells eventually succumb. *OXCT1* deletion downregulates stem markers and, interestingly, vascular recruitment genes. These results extended to our orthotopic tumor model where GR-OXCT1KO cells were less tumorigenic compared with the parental and 5637GR counterparts (Figures 6 and 7). The gemcitabine-resistant tumors seemed to recruit more vascularity than their isogenic counterparts, potentially due to the expression of the OVOL1/OVOL2 common target genes claudin-4 (*CLDN4*), *S100A14*, and metastasis associated with colon cancer 1 (*MACC1*) (56–58). In the context of gemcitabine, the 5637GR bladder tumors had elevated expression of the OVOL1 target genes, including *SOX2* and *SOX9*, in a OXCT1-dependent manner, resulting in greater mitotic index (Figure 8). Therefore, targeting OXCT1 can be beneficial for gemcitabine sensitization, not only by restricting energy and nucleotide precursors (required for DNA repair) but also by promoting epithelial cell fate through OVOL1 activity. The translocation of OVOL1 to the cytoplasm allows for shifting the signaling axis toward dedifferentiation and stemness (59). The discovery of OXCT1 and OVOL1 as metabolic and transcriptional regulators of gemcitabine sensitivity has broader implications in other gemcitabine-treated cancer types.

## Methods

### Sex as a biological variable.

For all the animal studies, male mice were used. This decision was made considering that 75% of the patients who suffer from BCa are male. Nevertheless, our results are expected to be relevant to all sexes.

### Data acquisition and analysis.

Gene expression and survival data for all data sets was obtained from the Gene Expression Omnibus (GEO, RRID:SCR_005012). The datasets used were: The Cancer Genome Atlas (TCGA), Mattias Höglund, Markus Riester, and Peter C. Black (22–24, 60). Data were analyzed using the median split and optimal cutoff approach to determine expression level of *OXCT1* and *HMGCS2*. To find the optimal cutoff value, we plotted the change of hazard ratio and 95% CI according to the cutoff value. Gene expression by staging was obtained from R2 (http://r2.amc.nl).

### RNA-Seq and MRA.

The 5637P and gemcitabine-resistant single cell clones were selected for RNA-Seq analysis. These were cultured until confluent, and RNA was extracted using the Qiagen RNeasy Kit with in-column DNase digestion. Sequencing was performed by Novogene on an Illumina NovoSeq 6000 platform. The data were analyzed using DESeq2 (RRID:SCR_000154) R package (v. 3.3) to identify differentially expressed genes (DEGs), enrichment analysis, and Gene Ontology biological processes. To identify key TFs, we performed a master regulator analysis. For this, we used DoRothEA regulon (41). Data for TF-target interaction information and significance of the TF were assessed using Fisher’s exact test and random permutation test. Target genes for individual TFs were identified from the DEGs, which were randomly sampled from the whole genome. This was repeated 10,000 times to generate an empirical null hypothesis of overlapping genes with DEGs. Finally, TFs with both Fisher’s exact test *P* < 0.05 and random permutation *P* < 0.05 were selected as key TFs for the regulation of the transcriptional changes.

### Gene silencing.

For stable Cas9-expressing cells, we used a lentivirus transfection method. We cotransfected 293T cells with lenti Cas9-Blast (Addgene, 52962), pCMV-dR8.91 (Addgene, 8455), and pCMV-VSVG (Addgene, 8454) following the BioT specifications (Bioland Scientific LLC, B01-01). To ensure 1 Cas9 infection per cell, we selected a viral titer that gave us a 10% infection efficiency. Briefly, virus was added to 5637 cells in suspension at different ratios including an uninfected control. Polybrene was added at 8 μg/mL to improve transduction. Two days after infection, we selected Cas9^+^ cells with blasticidin at 10 μg/mL while the cells were in suspension. The ratio that provided 10% infection rate was chosen for the experiments. *OXCT1* gRNAs were designed in the Synthego guide generating tool inserted in a gBlock and transfected as a pool in stable Cas9-expressing cells. Finally, OXCT1-KO lines were derived from stable single cell clones.

### Metabolic flux and metabolomics.

OCR was measured with the Seahorse Bioscience XF24 extracellular flux analyzer (Agilent). The cells were seeded to ensure 90% confluency for the assay. The cells were incubated in the assay medium for 1 hour at 37°C in a CO_2_-free incubator for temperature and pH equilibration. To determine ATP production, oligomycin (a complex V inhibitor) was used. To measure maximal respiration, FCCP (a strong uncoupler) was added; to completely shut down mitochondrial function and determine nonmitochondrial respiration, rotenone/antimycin (Complex I and III inhibitors respectively) were given (103015-100, Agilent). Initial assays were performed to optimize cell number, FCCP concentration, and oligomycin concentration (data not shown). Gemcitabine (0.1 μM) was used to measure acute treatment effects. Results were normalized to cell number using crystal violet staining and analyzed using 1-way ANOVA at a 95% CI (*n* = 5–7). Metabolomic profiling was performed on 5637P, 5637GR, and GR-OXCT1KO cells. Cells were treated for 72 hours with PBS or 0.01 μM gemcitabine and were at 70% confluency at the time of extraction. Metabolite extraction and liquid chromatography–MS (LC-MS) analysis were performed as previously described. Briefly, cells were washed with ice-cold 150 mM ammonium acetate (pH 7.3) and extracted in 80% methanol. Using a Vanquish Flex UPLC (Thermo Fisher Scientific), metabolites were separated on a Phenomenex Luna 3um NH2 100A (150 × 2.0 mm) column with mobile phases A (5 mM NH4AcO pH 9.9) and B (acetonitrile), detected in full scan mode with a Thermo Scientific Q Exactive mass spectrometer using polarity switching (+3.5 kV/−3.5 kV). Maven v8.1.27.11 was used to quantify the targeted metabolites by top area using expected retention time and accurate mass measurements (<5 ppm). Data analysis was performed using in-house R scripts (61).

### Multi-omics analysis.

Significant transcriptomics and metabolomics results were combined using Metaboanalyst 6.0 joint-pathway analysis (38). Briefly, only the significant DEGs, metabolites, and fold changes in 5637GR versus 5637P were selected for KEGG Application Programming Interface (API) combining metabolic and gene regulatory pathways. All gene symbols or open reading frames (ORFs) that did not have a match in the database were removed from the query during the mapping. To determine the number of pathways, the topology analysis was done prioritizing “degree centrality”. This function considers the number of links that connect to a pathway or node. To determine statistical significance, the proportion of *P* values and pathway impact corresponding to transcriptomics and metabolomics was done in an unweighted manner paying equal consideration to each enrichment analysis.

### Mouse models.

SCID mice (7- to 8-week-old male) were housed and maintained in a pathogen-free environment at the Cedars-Sinai animal facility. Animals received food and water ad libitum with a 12-hour light cycle. To generate tumors, we orthotopically injected 5 × 10^6^ isogenic 5637 cells (55 μL in neutralized rat tail collagen) in the bladder wall. Tumor growth was monitored every 2 weeks for 8 weeks after implantation via palpation. Tumor size before and after treatment was measured by ultrasound imaging as described by Patel et al. (62). Gemcitabine treatment (15 mg/kg) was administered every 3 days. Pre- and posttreatment μCT imaging was performed with iopamidol contrast agent at the Cedars-Sinai Research Imaging Core Facility (63). Harvested tumor mass was measured using an analytic balance and volume estimated by caliper measurements. We analyzed the results using 1-way ANOVA at a 95 % CI.

### Tissue analysis.

Tumor samples were collected and divided for snap freezing and fixed in 4% paraformaldehyde. The fixed tissue was processed, embedded in paraffin, and cut in 5 μm sections for histologic analysis. The frozen tissue was used to isolate RNA for gene expression assays. Histological examination included H&E staining and IHC localization. For IHC, deparaffinized tissues were treated with Antigen Unmasking Solution (1:100; H-3300-250, Vector Laboratories) followed by quenching of endogenous peroxidase with 3% v/v H_2_O_2_. SOX2 (1:200, 23064S Cell Signaling Technology), SOX9 (1:200; 82630, Cell Signaling Technology), phosphorylated histone H3 (1:200; 9701S, Cell Signaling Technology), and CD31 (1:100; ab28364, Abcam) were localized with respective primary antibodies. TUNEL staining was performed by manufacturer suggestion (EMD Millipore). All IHC was detected by the appropriate Envision system HRP-conjugated secondary antibodies (K400111-2, K400311-2, Agilent) according to the manufacturer’s instructions. Hematoxylin was used as a nuclear counterstain. Staining quantification was performed blinded to the treatment groups. The percentage of positive stained areas (e.g., vasculature, cytoplasm) or positively stained nuclei number per tumor field were quantified. Statistical analysis was done using 1-way ANOVA at 95 % CI with *n* = 5 representative areas.

### Tissue culture.

BCa cell lines 5637 and UM-UC3 were obtained from the American Type Culture Collection (ATCC). T-24 and RT4 cell lines were provided by Ruoxiang Wang and Leland Chung (Cedars-Sinai Medical Center, Los Angeles, California, USA). We maintained all cells in RPMI-1640 medium (Hyclone) supplemented with 10 % FBS (Hyclone) and Gentamicin (10 μg/mL; Thermo Fisher Scientific) at 37°C and 5% CO_2_ in a humidified incubator. The 5637GR cells were generated with a continuous exposure to gemcitabine. Starting from 1 nM gemcitabine, 5637 cells were treated in 72-hour intervals followed by a 72-hour recovery period. The gemcitabine concentration was increased once the viability after treatment was 80%. Increased drug exposure continued until the cells were resistant to 100 μM gemcitabine and considered stable. Resistant cells were maintained in culture without gemcitabine to have proper untreated controls.

### Wound healing assay.

The 5637 cells (2 × 10^5^ cells/mL) were grown until confluent in 12-well tissue culture plates. With a 200 μL pipette tip, a scratch wound was made across the center of the cell monolayer. Cells were photographed using a Nikon Eclipse TS100 microscope (Nikon) at 0, 6, 9, 12, and 24 hours at a 4× magnification. A total of 10 distance measurements within each wound were analyzed using Image Pro Plus Software. The differences in wound closure were normalized and compared with the control using 1-way ANOVA at a 95 % CI. All experiments were performed in triplicate, each with 3 wells per group.

### Sphere formation assay.

To study effect of OXCT1 on stemness, we performed a hanging drop sphere formation assay. Isogenic 5637 lines were plated on the lid of a 100 mm petri dish containing 15 mL of PBS. Each 30 μL droplet contained 6 × 10^4^ cells/mL. Cells were resuspended using a needle to ensure single-cell suspension. Droplets were arranged in a quadrant allowing 16 droplets per petri dish to ensure even distribution and avoid evaporation. Pictures were taken at day 3 and day 6 at 10× magnification using an Olympus FSX-100 microscope.

### Flow cytometry.

Cells were treated with gemcitabine at the IC_50_ dose for 5637P cells (0.01 μM) for 72 hours. Cells were detached by accutase (Thermo Fisher Scientific), washed, and resuspended in FACS buffer (1% BSA in PBS). Antibodies CD44 (PE Cy7: FL3; 60-0441, Tonbo Biosciences), CD36 (PE: FL2; 561821, BD Biosciences) were used at (1:100) in FACS buffer. Cells were fixed in 1% PFA in PBS, run on the Accuri C6 flow cytometer (BD Biosciences), and analyzed using FlowJo (RRID:SCR_008520 FlowJo LLC, BD Biosciences).

### Western blotting.

The 5637 cells were maintained in culture and treated for 72 hours with gemcitabine at IC_50_ or PBS. For nuclear fractionation, the cytoplasmic fraction was extracted with hypotonic buffer (20 mM Tris-HCl [Thermo Fisher Scientific], 10 mM KCl [Thermo Fisher Scientific], 2 mM MgCl_2_ [Sigma-Aldrich], 1 mM EGTA [Sigma-Aldrich], 0.1 % IGEPAL [Alpha Aesar] [pH 7.4]), and the nuclear fraction was extracted with isotonic buffer (20 mM Tris-HCl, 150 mM KCl, 2 mM MgCl_2_, 1 mM EGTA, 0.3 % IGEPAL [pH 7.4]). For Western blots, 30 μg of protein were assayed in nuclear and cytoplasmic fractions. The following antibodies were used: OXCT1 (HPA061425, Sigma-Aldrich), HMGCS2 (20940S, Cell Signaling Technology), CPT1A (12252S, Cell Signaling), PPARγ (sc-7273, Santa Cruz Biotechnology Inc.) OVOL1 (PA5-41480, Thermo Fisher Scientific), OVOL2 (NB030227, Novus Biological), β-actin (sc-47778, Santa Cruz Biotechnology Inc.), Laminb1 (sc-377000, Santa Cruz Biotechnology Inc.), and Tom20 (sc-17764, Santa Cruz Biotechnology Inc.). Experiments were performed in triplicate and densitometry was calculated with ImageJ (NIH).

### Statistics.

All experiments were performed with a minimum of 3 biological replicates. Paired comparisons were performed with a 2-tailed *t* test at a 95% CI. Larger group studies were analyzed with 1-way ANOVA at a 95% CI and Tukey multiple comparisons test. For all studies, significance is considered at *P* < 0.05.

### Study approval.

All animal studies were performed with IACUC approval at Cedars-Sinai Medical Center.

### Data availability.

Sequencing data are available at GEO (RRID:SCR_005012), ID: GSE280095. Values for all data points in graphs are reported in the Supporting Data Values file.

## Author contributions

Idea development was contributed by KRR and NAB. Experimental design was contributed by KRR and NAB. Experiment execution was contributed by KRR, SB, and GG. Data collection was contributed by KRR, SB, GG, CH, and AH. Data analysis was contributed by KRR, SY, MK, and JTH. Project supervision was contributed by NAB. Technical review was contributed by KSC. All authors contributed to the manuscript preparation and review.

## Supplementary Material

Supplemental data

Unedited blot and gel images

Supporting data values

## Version 1 11/07/2024

In-Press Preview

## Version 2 12/20/2024

Electronic publication
