# Supplementary material for: Targeting ketone body metabolism in mitigating gemcitabine resistance
Source: JCI Insight. 2024 Dec 20;9(24):e177840. doi: 10.1172/jci.insight.177840 (PMC11665555; doi:10.1172/jci.insight.177840)
Supplement: Supplemental data [file jciinsight-9-177840-s043.pdf]

Supplementary Material

Title: Targeting Ketone Body Metabolism in Mitigating Gemcitabine Resistance

Authors:

Krizia Rohena Rivera[1,2], Sungyong You[2,3,4], Minhyung Kim[3,], Sandrine Billet[1,2], Johanna ten Hoeve[5], Gabrielle Gonzales[1,2], Chengqun Huang[1], Ashley Heard[1,2], Keith Syson Chan[6], Neil A. Bhowmick[1,2,7]*

Affiliations:

[1] Cedars-Sinai Medical Center, Los Angeles CA, USA

[2] Samuel Oschin Comprehensive Cancer Institute, Los Angeles CA, USA

[3] Department of Urology, Cedars-Sinai Medical Center, USA

[4] Department of Computational Biomedicine, Cedars-Sinai Medical Center, USA

[5] UCLA Metabolomics Center, Department of Molecular & Medical Pharmacology, University of California Los Angeles, Los Angeles CA, USA

[6] Department of Urology and Neal Cancer Center, Houston Methodist Research Institute, Houston TX, USA

[7] Department of Research, VA Greater Los Angeles Healthcare System, Los Angeles CA, USA

*** Corresponding author:** Neil A. Bhowmick

neil.bhowmick@cshs.org

Supplementary Figures:

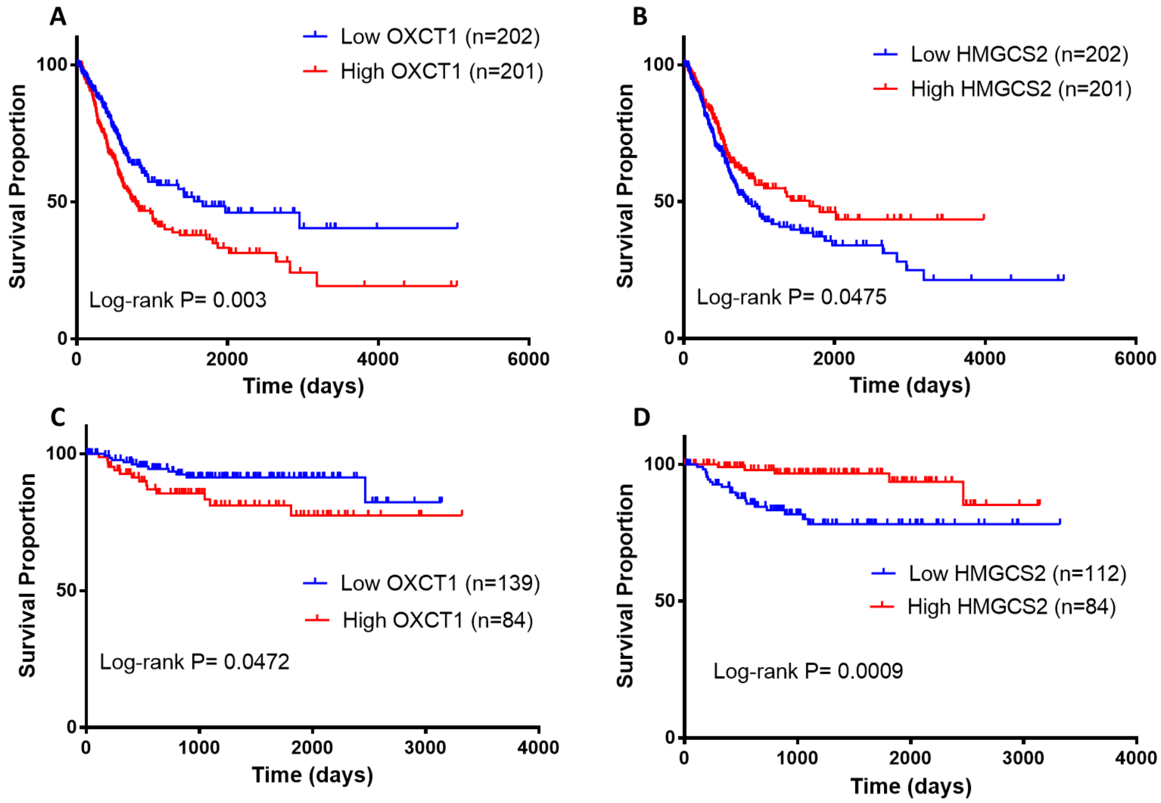

Fig. S1: Association of OXCT1 and HMGCS2 expression with survival in the TCGA and Höglund dataset. Data and analysis were obtained in R2 genomics. The expression cutoff was determined using the median split. **A)** TCGA, OXCT1 expression and overall survival. High OXCT1 associates with poor survival p = 0.003. **B)** TCGA, HMGCS2 expression and overall survival. High HMGCS2 is associated with better outcome p = 0.0475. **C)** Höglund, High OXCT1 associates with poor survival p = 0.0472. **D)** Höglund, High HMGCS2 is associated with better overall survival p = 0.0009.

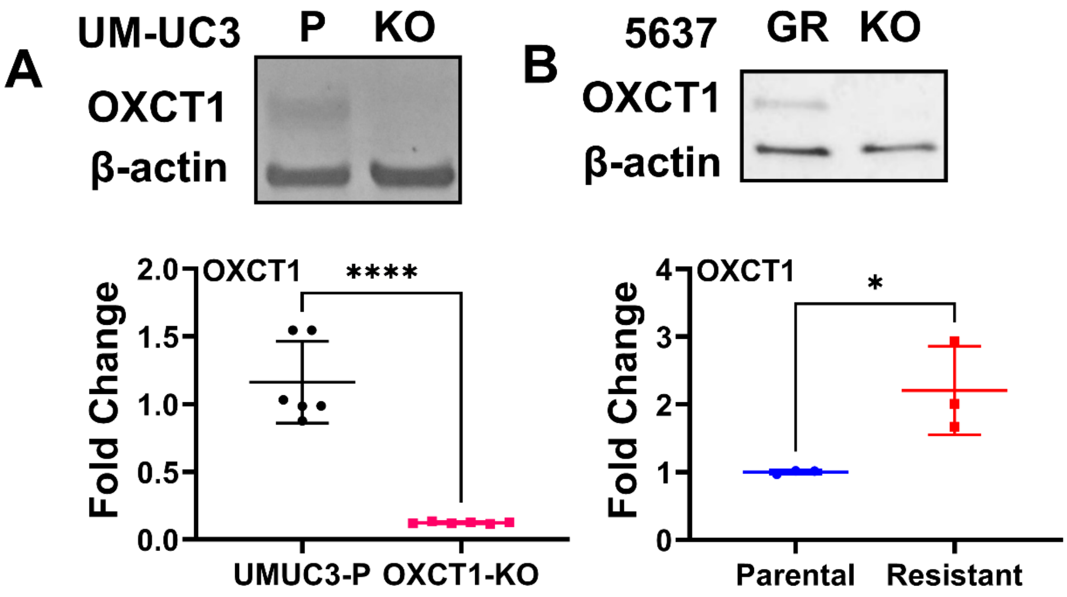

Fig. S2. OXCT1 expression in CRISPR/CAS9 knockout and resistant cells. A) UM-UC3 OXCT1 knockout confirmed by qPCR (Two tailed T-test, 95 % CI, ****$P < 0.0001$) and Western Blot. **B)** Western Blot of 5637GR and GR OXCT1 KO, OXCT1 expression increases in 5637GR confirmed by qPCR (Two tailed T-test, 95 % CI, *$P < 0.05$).

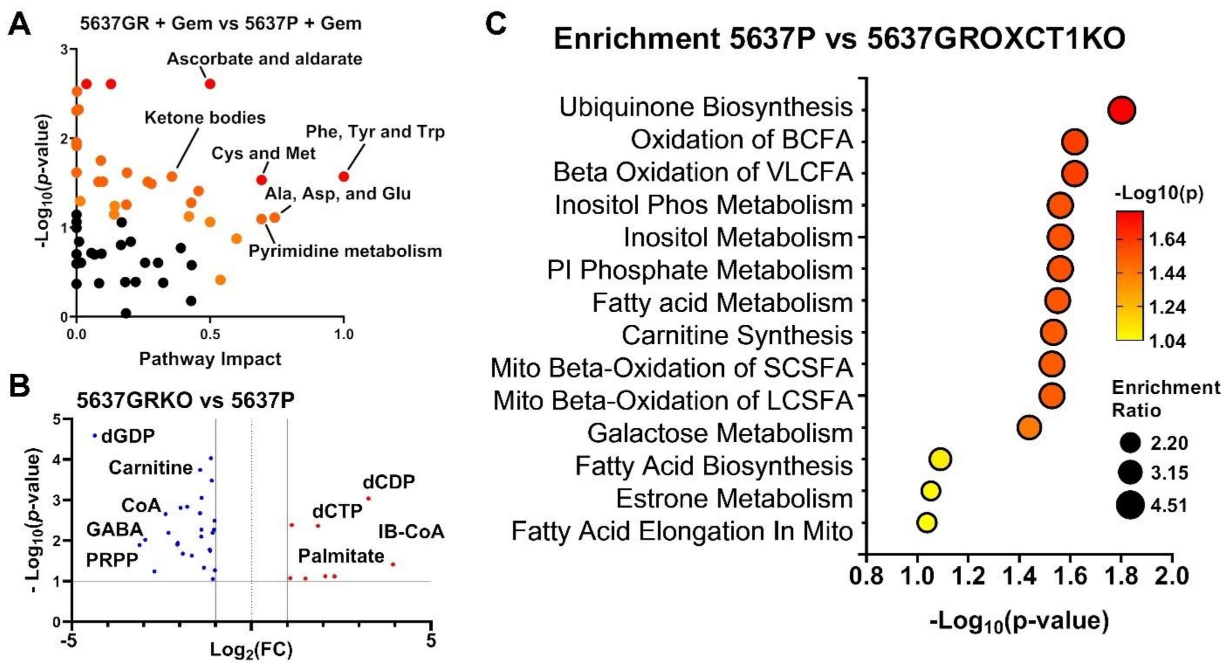

Fig. S3: Metabolomic pathway enrichment analysis. A) The pathway impact and statistical significance for the indicated metabolic pathways affected by gemcitabine treatment 5637GR vs 5637P. The details are described in Table S3. **B)** Volcano plot comparing 5637GROXCT1KO and 5637P to highlight the key metabolites associated with OXCT1 that may affect gemcitabine sensitivity. Results show a significant decrease in carnitine, PRPP and GABA which suggest a decrease on oxidative processes. **C)** Metabolite enrichment analysis for 5637P and 5637GROXCT1KO shows oxidative processes to be the most significantly different upon gemcitabine stress. Knocking out OXCT1 significantly reduces oxidation while parental cells can still adapt their metabolism and acquire resistance.

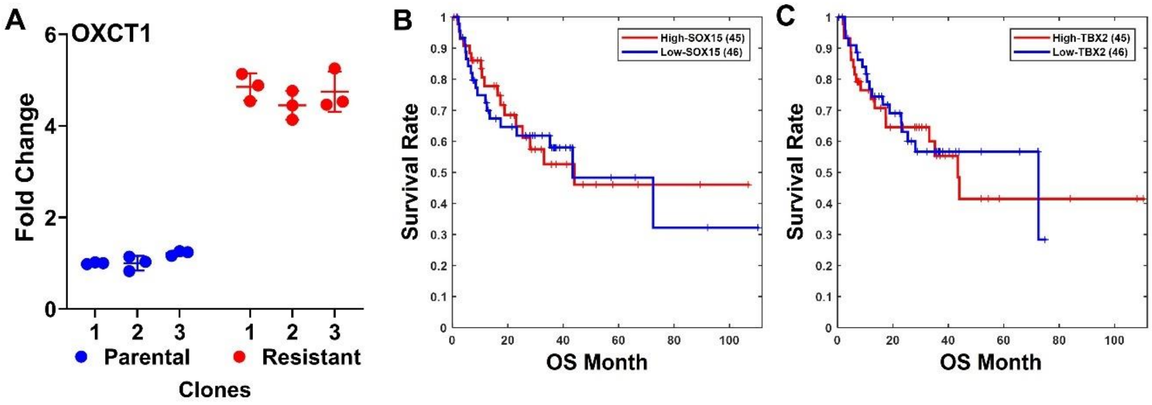

Fig. S4: Independent Cell Clones and Downstream targets of SOX15 and TBX2 in the Peter C. Black dataset. A) Independent parental and resistant single cell clones maintain OXCT1 expression pattern. **B)** After MRA, the expression of the SOX15 and TBX2 downstream targets was analyzed in the pre-treatment and Gem-Cis cohort of the Peter C. Black dataset using the median as the expression cutoff (n = 91). Kaplan-Meier Plot of SOX15 downstream targets HR = 0.85, 95% CI [0.44-1.66], p = 0.6424. **C)** Kaplan-Meier Plot of TBX2 downstream targets HR = 1.97, 95% CI [0.99-3.91], p = 0.0535.

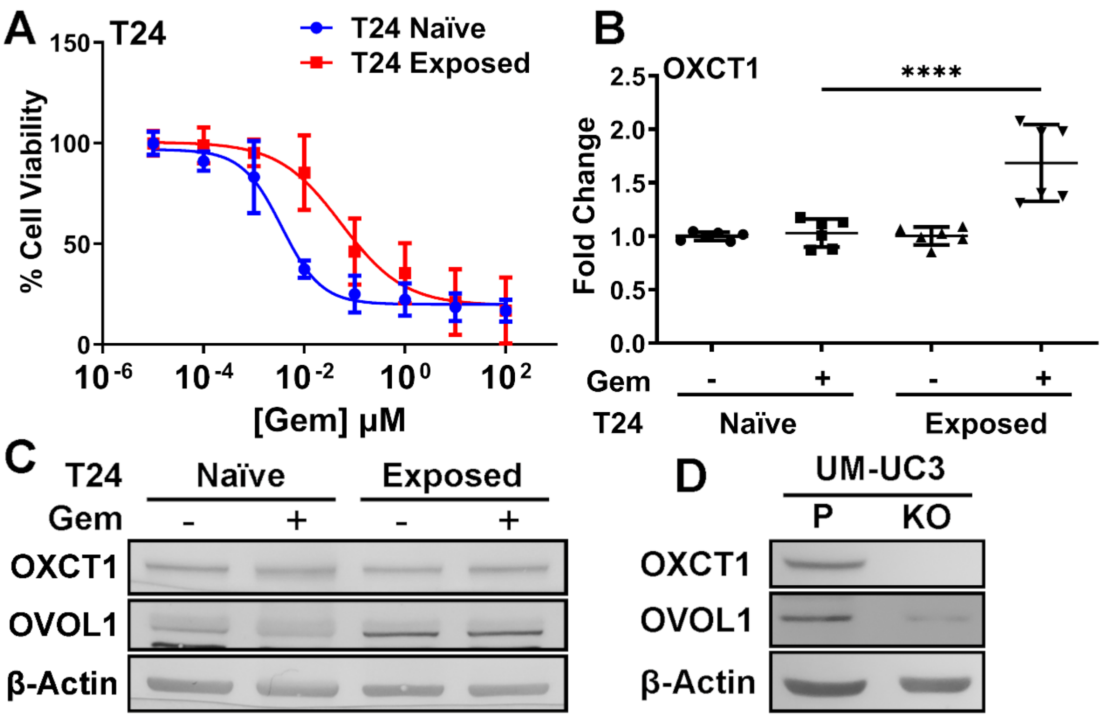

Fig. S5: OXCT1 affects resistance in T24 and UM-UC3 cell lines. A) T24 exposed cells were treated with gemcitabine at IC_{50} concentration for 4 passages and allowed a washout period. Data shows that T24 cells developed resistance to the drug T24 naïve (IC_{50} = 0.0037395) and exposed (IC_{50} = 0.066055). **B)** OXCT1 expression in naïve and gemcitabine exposed T24 cells. Statistical analysis done with one-way ANOVA 95 % CI, $p < 0.0001$. **C)** Cytoplasmic fraction western blot of T24 and T24E cells showing OVOL1 and OXCT1 expression. Resistant cells have increased OVOL1 in the cytoplasmic fraction. **D)** Cytoplasmic fraction western blot of UM-UC3 cells and UM-UC3 OXCT1KO showing the OVOL1 is reduced in the cytoplasmic fraction.

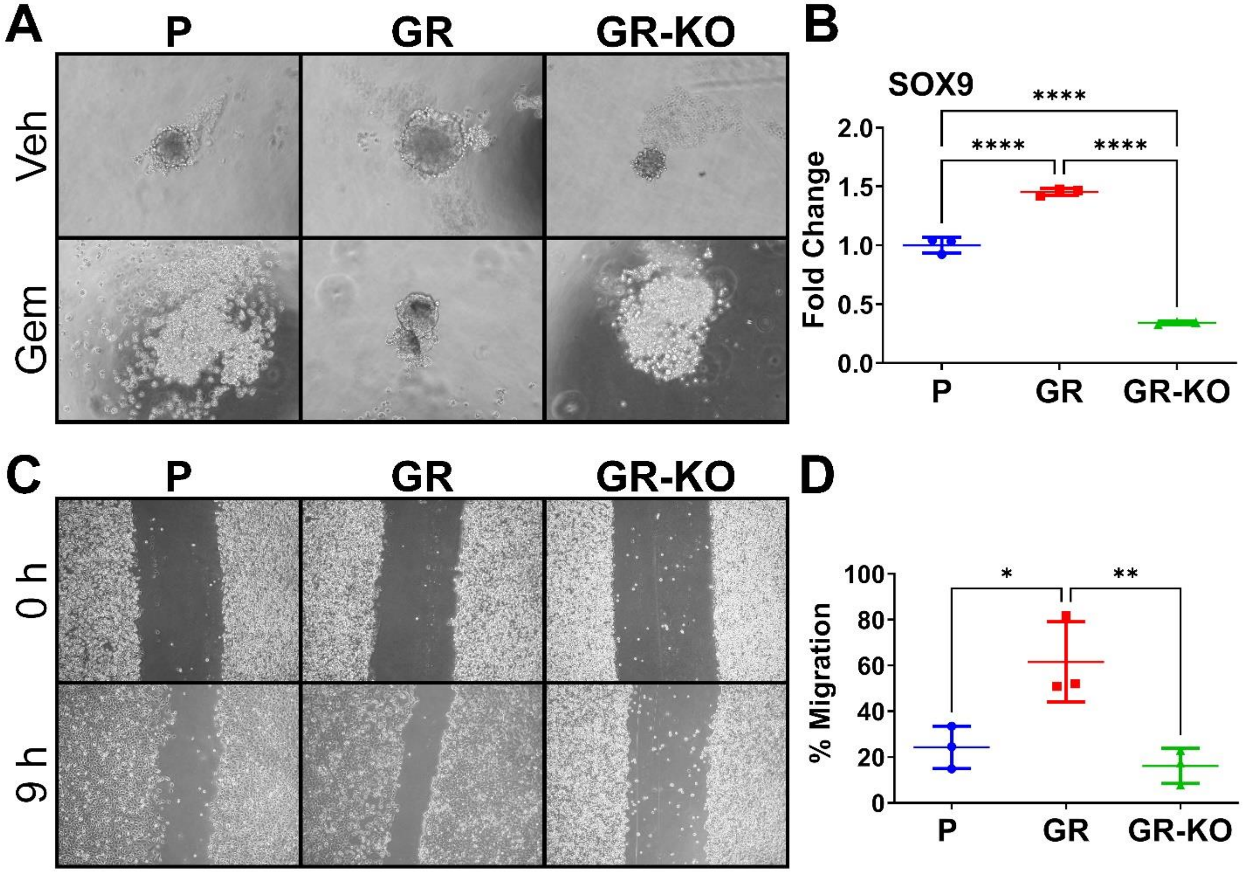

Fig. S6: OXCT1 affects stemness and cell motility. A) Hanging drop sphere formation assay. Overall knocking out OXCT1 produced smaller spheres. Exposing the spheres to gemcitabine completely disrupted sphere integrity of 5637P and 5637GROXCT1KO. **B)** qPCR analysis for stem marker SOX9 (One-way ANOVA, 95 % CI, ****$P < 0.0001$). **C)** Cell migration was studied using a wound healing assay. Cells were cultured until they formed a confluent monolayer, and a wound was created with a pipette tip. At 9h there was a significant difference in the gap closure compared to the parental line (One-way ANOVA, 95 % CI, *$P < 0.05$, **$P < 0.01$)

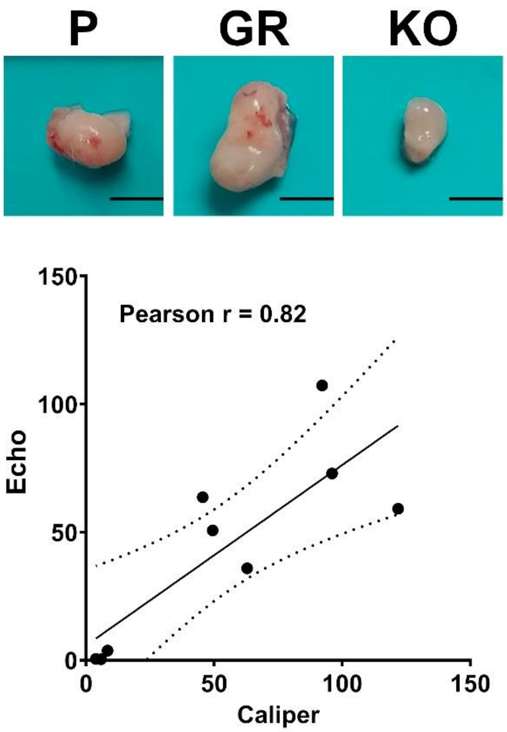

Fig. S7: Tissue images and validation of ultrasound volume measurements. Representative images of tumors at endpoint (scale = 5 mm). Tumor volume was calculated from ultrasound (Echo) imaging following the method described by Patel et al. The volume was calculated with the formula π/6 x length x width from the anterior-posterior plane. These results were compared to the manual caliper measurements and calculation using ellipsoid volume formula. Pearson correlation validates our assay with r = 0.82.

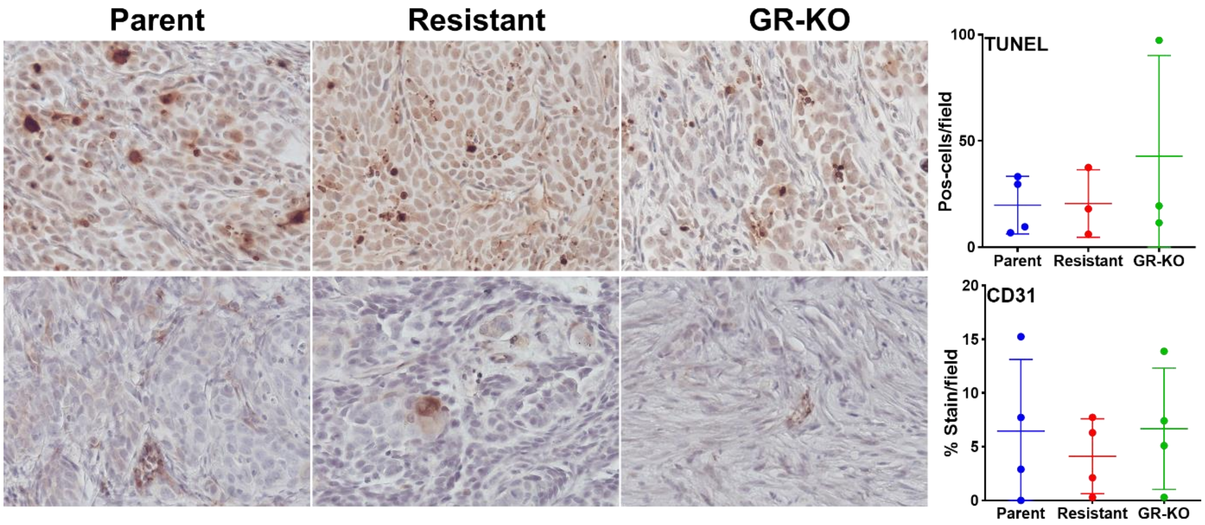

Fig. S8: CD31 and TUNEL staining in gemcitabine treated tumors. Representative microscope images at 20x and the respective quantification. We did not observe any statistical difference in staining (One-way ANOVA at 95 %, CI).

Supplementary Tables:

Table S1: ANOVA Post-hoc analysis of all samples

Metabolites	F value	P value
CDP-ethanolamine	109.87	2.51E-12
CDP-choline	83.795	2.29E-11
Phosphoethanolamine	78.721	3.80E-11
Orotidine	54.14	7.62E-10
Inositol	39.941	8.24E-09
2-HG	36.08	1.80E-08
S-Adenosyl-L-methionine	32.043	4.44E-08
Carnitine	28.95	9.53E-08
Acetylcarnitine	25.636	2.34E-07
Glycerol-3-phosphate	22.339	6.39E-07
dATP	21.684	7.91E-07
Dihydroorotate	19.799	1.51E-06
NADPH	19.636	1.60E-06
dCDP	18.552	2.39E-06
Phenylalanine	18.441	2.49E-06
Tryptophan	16.606	5.13E-06
UDP-N-acetylglucosamine	15.348	8.74E-06
Malate	14.996	1.02E-05
Serine	14.829	1.10E-05
Lysine	14.2	1.47E-05
Fumarate	13.98	1.63E-05
Tyrosine	12.759	2.95E-05
Sarcosine	12.25	3.83E-05
UDP-glucose	12.158	4.02E-05
Hypoxanthine	11.566	5.51E-05
Phosphocreatine	11.317	6.31E-05
Uridine	10.809	8.39E-05
Creatine	10.39	0.000107
dCTP	10.258	0.000115
Gluconic acid	10.215	0.000118
Adenine	10.149	0.000123
Choline	9.9592	0.000138
GABA	9.9104	0.000142
Acetylcholine	9.7856	0.000153
Cytidine	9.5083	0.000182
CMP	9.4516	0.000188
Histidine	9.1464	0.000229
Acetyl-CoA	9.0516	0.000243
Inosine	7.9408	0.000512
2-Aminobutyrate	7.9183	0.00052
Glucose	7.8479	0.000547

dGDP	7.8143	0.00056
Pantothenate	7.6415	0.000633
Cytosine	7.4199	0.000744
dTTP	7.4164	0.000746
NAD+	7.3961	0.000757
CoA	7.1574	0.000903
Adenosine	7.0992	0.000943
Threonine	7.0378	0.000988
Glucosamine-6-phosphate	6.7986	0.001186
Methionine	6.5771	0.001409
Phosphoribosyl pyrophosphate	6.5639	0.001424
6-Phosphogluconate	6.4862	0.001514
Carbamoylaspartate	6.0257	0.002198
Guanosine	5.8421	0.002561
Aconitate	5.6083	0.003124
Deoxythymidine	5.5854	0.003186
Ribose-5-phosphate	5.5807	0.003199
Thymine	5.5784	0.003205
Proline	5.4742	0.003508
Citrate	5.2579	0.004242
4-Hydroxyphenyllactate	5.1421	0.004705
Deoxycytidine	5.1218	0.004792
Cystine	5.1166	0.004814
Phosphocholine	5.0073	0.005315
CTP	4.8883	0.005926
Aspartate	4.7109	0.006989
GTP	4.5418	0.008201
Leucine-Isoleucine	4.3992	0.009405
Sedoheptulose-7-phosphate	4.1839	0.011609
Succinate	3.9943	0.01403
Deoxyuridine	3.9249	0.015051
CDP	3.7498	0.018011
Glycerol	3.7202	0.018571
Folate	3.7154	0.018664
N-Acetylglucosamine-6-phosphate	3.6972	0.01902
Succinyl-CoA	3.4727	0.02409

Table S2: Metabolomic pathway analysis 5637 resistant vs parental untreated

Pathway	Total Cmpd	Hits	Raw p	LOG10(p)	Holm adjust	FDR	Impact
Phenylalanine, tyrosine and tryptophan biosynthesis	4	2	0.019458	1.7109	1	0.18161	1
Alanine, aspartate and glutamate metabolism	28	13	0.30983	0.50887	1	0.55621	0.74119
Pyrimidine metabolism	39	22	0.3257	0.48718	1	0.55621	0.69248
Cysteine and methionine metabolism	33	10	0.027401	1.5622	1	0.19181	0.69232
Arginine biosynthesis	14	9	0.30799	0.51146	1	0.55621	0.59898
Pentose phosphate pathway	22	7	0.04343	1.3622	1	0.27023	0.53875
Ascorbate and aldarate metabolism	8	3	0.013548	1.8681	0.75812	0.18161	0.5
D-Glutamine and D-glutamate metabolism	6	3	0.29517	0.52993	1	0.55621	0.5
Glycine, serine and threonine metabolism	33	10	0.77072	0.1131	1	0.84628	0.4568
Glutathione metabolism	28	10	0.6306	0.20024	1	0.76769	0.43063
Arginine and proline metabolism	38	9	0.39369	0.40485	1	0.61233	0.42925
Nicotinate and nicotinamide metabolism	15	4	0.72582	0.13917	1	0.82539	0.42895
Purine metabolism	65	21	0.43154	0.36498	1	0.61965	0.42127
Citrate cycle (TCA cycle)	20	9	0.14799	0.82977	1	0.55621	0.39047
Phenylalanine metabolism	10	2	0.019458	1.7109	1	0.18161	0.35714
Glycolysis / Gluconeogenesis	26	6	0.70271	0.15322	1	0.82539	0.32428
Fatty acid degradation	39	3	0.3377	0.47147	1	0.55621	0.30496
Glycerolipid metabolism	16	2	0.20626	0.68559	1	0.55621	0.28037
Synthesis and degradation of ketone bodies	5	2	0.84603	0.072615	1	0.86141	0.26667
Fatty acid elongation	39	2	0.3377	0.47147	1	0.55621	0.25661
Histidine metabolism	16	3	0.31668	0.49938	1	0.55621	0.22131
Pentose and glucuronate interconversions	18	2	0.61602	0.21041	1	0.7666	0.20312
Tyrosine metabolism	42	3	0.06878	1.1625	1	0.35322	0.18898
Glycerophospholipid metabolism	36	7	0.40563	0.39187	1	0.61233	0.18677
Pyruvate metabolism	22	5	0.73696	0.13256	1	0.82539	0.18507
Pantothenate and CoA biosynthesis	19	5	0.84247	0.074444	1	0.86141	0.18214
Glyoxylate and dicarboxylate metabolism	32	8	0.2594	0.58604	1	0.55621	0.16932
Amino sugar and nucleotide sugar metabolism	37	6	0.18082	0.74275	1	0.55621	0.16627
Tryptophan metabolism	41	3	0.025186	1.5988	1	0.19181	0.14305
Lysine degradation	25	3	0.12712	0.89578	1	0.5476	0.14085

Inositol phosphate metabolism	30	4	0.013538	1.8684	0.75812	0.18161	0.12939
Valine, leucine and isoleucine degradation	40	4	0.84448	0.073411	1	0.86141	0.09836
Propanoate metabolism	23	4	0.17071	0.76773	1	0.55621	0.09391
Folate biosynthesis	27	2	0.93592	0.028763	1	0.93592	0.09111
Butanoate metabolism	15	7	0.29284	0.53337	1	0.55621	0.08466
Terpenoid backbone biosynthesis	18	2	0.84603	0.072615	1	0.86141	0.08254
Fructose and mannose metabolism	20	2	0.26376	0.5788	1	0.55621	0.06695
beta-Alanine metabolism	21	4	0.61227	0.21306	1	0.7666	0.05597
Phosphatidylinositol signaling system	28	1	0.013538	1.8684	0.75812	0.18161	0.03736
Fatty acid biosynthesis	47	2	0.3377	0.47147	1	0.55621	0.01686
Sphingolipid metabolism	21	1	0.50121	0.29998	1	0.66827	0.0142
Starch and sucrose metabolism	18	1	0.616	0.21042	1	0.7666	0.00974
Primary bile acid biosynthesis	46	1	0.15953	0.79717	1	0.55621	0.00758
Galactose metabolism	27	4	0.014156	1.8491	0.75812	0.18161	0.00228
Ubiquinone and other terpenoid-quinone biosynthesis	9	1	0.069383	1.1587	1	0.35322	0
Biotin metabolism	10	1	0.12402	0.9065	1	0.5476	0
Selenocompound metabolism	20	1	0.20712	0.68378	1	0.55621	0
Porphyrin and chlorophyll metabolism	30	2	0.28845	0.53992	1	0.55621	0
Nitrogen metabolism	6	2	0.29517	0.52993	1	0.55621	0
Aminoacyl-tRNA biosynthesis	48	16	0.30464	0.51621	1	0.55621	0
Biosynthesis of unsaturated fatty acids	36	1	0.33769	0.47148	1	0.55621	0
Valine, leucine and isoleucine biosynthesis	8	2	0.38468	0.4149	1	0.61233	0
One carbon pool by folate	9	1	0.41551	0.38142	1	0.61233	0
Taurine and hypotaurine metabolism	8	1	0.4676	0.33013	1	0.63867	0
Thiamine metabolism	7	1	0.4676	0.33013	1	0.63867	0
Mannose type O-glycan biosynthesis	17	1	0.73156	0.13575	1	0.82539	0

Table S3: Metabolic pathway analysis 5637 resistant vs parental gemcitabine treated.

Pathway	Total Cmpd	Hits	Raw p	LOG10(p)	Holm adjust	FDR	Impact
Phenylalanine, tyrosine and tryptophan biosynthesis	4	2	0.026853	1.571	1	0.10068	1
Alanine, aspartate and glutamate metabolism	28	13	0.077438	1.111	1	0.15819	0.74119
Pyrimidine metabolism	39	22	0.080509	1.0942	1	0.15819	0.69248
Cysteine and methionine metabolism	33	10	0.029357	1.5323	1	0.10068	0.69232
Arginine biosynthesis	14	9	0.13338	0.87493	1	0.22633	0.59898
Pentose phosphate pathway	22	7	0.38635	0.41302	1	0.44154	0.53875
Ascorbate and aldarate metabolism	8	3	0.00246	2.609	0.13777	0.041734	0.5
D-Glutamine and D-glutamate metabolism	6	3	0.086601	1.0625	1	0.15819	0.5
Glycine, serine and threonine metabolism	33	10	0.038887	1.4102	1	0.11461	0.4568
Glutathione metabolism	28	10	0.26407	0.57828	1	0.30808	0.43063
Arginine and proline metabolism	38	9	0.052815	1.2772	1	0.13935	0.42925
Nicotinate and nicotinamide metabolism	15	4	0.66584	0.17663	1	0.67795	0.42895
Purine metabolism	65	20	0.075113	1.1243	1	0.15819	0.42026
Citrate cycle (TCA cycle)	20	9	0.16967	0.7704	1	0.2568	0.39047
Phenylalanine metabolism	10	2	0.026853	1.571	1	0.10068	0.35714
Glycolysis / Gluconeogenesis	26	6	0.41683	0.38004	1	0.44528	0.32428
Fatty acid degradation	39	3	0.24823	0.60514	1	0.30407	0.30496
Glycerolipid metabolism	16	2	0.032363	1.49	1	0.10068	0.28037
Synthesis and degradation of ketone bodies	5	2	0.030699	1.5129	1	0.10068	0.26667
Fatty acid elongation	39	2	0.24825	0.60512	1	0.30407	0.25661
Histidine metabolism	16	3	0.40736	0.39002	1	0.44528	0.22131
Pentose and glucuronate interconversions	18	2	0.144	0.84163	1	0.23043	0.20312
Tyrosine metabolism	42	3	0.024332	1.6138	1	0.10068	0.18898
Glycerophospholipid metabolism	36	7	0.055479	1.2559	1	0.13935	0.18677
Pyruvate metabolism	22	5	0.91097	0.040495	1	0.91097	0.18507
Pantothenate and CoA biosynthesis	19	5	0.4088	0.38849	1	0.44528	0.18214
Glyoxylate and dicarboxylate metabolism	32	8	0.087567	1.0577	1	0.15819	0.16932
Amino sugar and nucleotide sugar metabolism	37	6	0.1568	0.80467	1	0.2439	0.16627
Tryptophan metabolism	41	3	0.057235	1.2423	1	0.13935	0.14305
Lysine degradation	25	3	0.071381	1.1464	1	0.15819	0.14085
Inositol phosphate metabolism	30	4	0.002463	2.6086	0.13777	0.041734	0.12939
Valine, leucine and isoleucine degradation	40	4	0.030601	1.5143	1	0.10068	0.09836

Propanoate metabolism	23	4	0.19639	0.70689	1	0.26819	0.09391
Folate biosynthesis	27	2	0.017705	1.7519	0.84986	0.10068	0.09111
Butanoate metabolism	15	7	0.42178	0.37491	1	0.44528	0.08466
Terpenoid backbone biosynthesis	18	2	0.030699	1.5129	1	0.10068	0.08254
Fructose and mannose metabolism	20	2	0.20114	0.6965	1	0.26819	0.06695
beta-Alanine metabolism	21	4	0.19275	0.715	1	0.26819	0.05597
Phosphatidylinositol signaling system	28	1	0.002463	2.6086	0.13777	0.041734	0.03736
Fatty acid biosynthesis	47	2	0.24825	0.60512	1	0.30407	0.01686
Sphingolipid metabolism	21	1	0.050732	1.2947	1	0.13935	0.0142
Starch and sucrose metabolism	18	1	0.14402	0.84159	1	0.23043	0.00974
Primary bile acid biosynthesis	46	1	0.00476	2.3224	0.24754	0.045886	0.00758
Galactose metabolism	27	4	0.002981	2.5256	0.15799	0.041734	0.00228
Selenocompound metabolism	20	1	0.004916	2.3084	0.25073	0.045886	0
Mannose type O-glycan biosynthesis	17	1	0.011132	1.9534	0.5566	0.085213	0
Valine, leucine and isoleucine biosynthesis	8	2	0.012173	1.9146	0.59649	0.085213	0
Ubiquinone and other terpenoid-quinone biosynthesis	9	1	0.024125	1.6175	1	0.10068	0
Biotin metabolism	10	1	0.071758	1.1441	1	0.15819	0
Nitrogen metabolism	6	2	0.086601	1.0625	1	0.15819	0
Aminoacyl-tRNA biosynthesis	48	16	0.10078	0.99661	1	0.17637	0
Taurine and hypotaurine metabolism	8	1	0.19889	0.70138	1	0.26819	0
Thiamine metabolism	7	1	0.19889	0.70138	1	0.26819	0
One carbon pool by folate	9	1	0.25386	0.59541	1	0.30407	0
Biosynthesis of unsaturated fatty acids	36	1	0.2552	0.59312	1	0.30407	0
Porphyrin and chlorophyll metabolism	30	2	0.42937	0.36716	1	0.44528	0

Table S4. Validation of the top differentially expressed genes by qPCR.

GENE NAME	GR (FC)	P-Value	GR-OXCT1-KO (FC)	P-Value
**UPREGULATED**				
**CPED1**	2.49	4.32E-07	0.85	1.29E-04
**C17IRF51**	1.47	1.37E-02	0.52	1.51E-02
**TBX2**	4.36	6.39E-07	0.92	2.66E-01
**HDDC2**	3.88	4.73E-06	0.11	8.35E-06
**PXDN**	1.23	1.98E-01	0.85	1.84E-01
**AADAC**	10.24	9.64E-08	1.33	3.14E-02
**TRNP1**	0.90	2.13E-01	0.43	9.80E-04
**ZNF667**	0.67	1.33E-01	0.31	1.81E-02
**DPY19L2P2**	1.00	9.80E-01	0.26	3.49E-06
**LINC00839**	1.47	8.51E-04	0.49	3.17E-04
**DOWNREGULATED**				
**ANXA6**	0.08	9.42E-08	0.13	8.88E-08
**ADH1C**	0.59	2.69E-02	0.74	9.58E-02
**TNNT3**	11.77	2.07E-03	3.74	2.99E-02
**PAD12**	1.38	1.43E-03	0.19	2.92E-06
**PELI2**	0.78	8.39E-03	0.56	1.03E-03
**TMTC1**	3.98	7.96E-05	0.54	1.46E-02
**OAS1**	0.01	2.21E-04	1.55	1.22E-02
**TP53L11**	0.09	8.71E-07	0.20	7.47E-06
**IGFBP3**	0.86	1.59E-05	0.12	4.77E-10

Table S5: Enrichment of all differentially expressed genes.

GeneName	5637-GR	5637-P	log2FoldChange	pvalue
C17orf51	112.5106354	17.25024	2.0341	4.66E-12
HDDC2	2541.807018	391.5148	1.9008	1.35E-09
ZNF667	49.87571049	2.705857	1.8748	5.68E-08
AADAC	100.103165	10.54552	1.8672	3.41E-08
LINC00839	37.79862737	1.41176	1.8584	7.39E-08
TBX2	279.0975573	57.34144	1.8488	6.46E-12
CPED1	173.3915173	36.96367	1.8271	5.23E-12
DPY19L2P2	174.7945794	26.90688	1.7588	7.27E-08
TRNP1	245.8664549	41.91389	1.7457	4.54E-08
PXDN	641.7221011	128.1653	1.7345	7.02E-09
BTBD19	150.9611666	22.73188	1.6888	4.06E-07
IL31RA	35.469595	2.032377	1.6337	2.16E-06
S100A14	151.1390115	29.86169	1.6111	4.10E-07
NAGS	256.0811593	51.36875	1.5398	2.11E-06
AC004696.2	43.10817887	4.046568	1.535	8.86E-06
DYSF	243.5441355	43.02387	1.5032	8.06E-06
RP11-64D22.2	60.29102608	9.48159	1.4917	1.22E-05
PCNPP3	90.0661735	5.872086	1.4843	1.45E-05
CTD-2021J15.2	112.013336	10.8871	1.4766	1.88E-05
MYH16	126.0064535	19.66565	1.4741	1.66E-05
CLDN3	94.57909408	12.88936	1.4682	2.05E-05
BSPRY	146.3360633	23.49343	1.4574	2.06E-05
TMC8	70.23684548	7.974947	1.4428	2.96E-05
RP11-64D22.5	50.23928782	8.938272	1.393	4.49E-05
TMEM200B	256.7113005	61.07554	1.3892	1.77E-05
SH2D5	670.6407332	201.0625	1.368	1.26E-06
FHIT	64.47792773	16.163	1.3646	1.96E-05
TRHDE	58.4131754	15.08145	1.3502	2.03E-05
CAMK2N2	298.7022127	82.87519	1.325	1.98E-05
RP11-332H18.4	40.7610205	9.398643	1.3076	8.99E-05
PGF	186.0203289	36.92723	1.3039	0.000138
CA10	81.44123833	13.82937	1.3032	0.000158
MMP17	465.0404685	112.3771	1.2978	9.23E-05
TRIM22	55.74874058	10.36822	1.2858	0.000186
SHOX2	68.35505215	18.68471	1.2824	5.58E-05
RGL3	1380.300726	475.4576	1.2731	1.04E-06
IGFBP7	202.4389577	62.89815	1.2725	1.86E-05
VEPH1	93.04546259	27.96512	1.2634	3.67E-05
IL6R	234.4412067	63.49938	1.263	9.65E-05
PKIB	77.87705294	8.247171	1.2509	0.00025
AC099342.1	79.56338764	19.21044	1.2473	0.000204
GPR176	391.048476	117.8428	1.2469	5.55E-05

CABP7	82.7521789	16.95584	1.2411	0.000298
MTMR8	27.70409611	3.674304	1.2374	0.00033
GCNT2	76.98774393	11.84421	1.2159	0.000425
ANTXR2	3834.196556	1296.28	1.2108	2.73E-05
CDH7	73.17358783	19.80944	1.208	0.00025
COL9A2	387.0464657	135.7944	1.1999	1.62E-05
RNF208	42.52162801	7.313402	1.1981	0.000524
PVRL4	87.70593523	23.00979	1.1893	0.000386
TMCC2	307.6253578	106.8125	1.185	3.63E-05
LINC00704	82.8810182	17.40992	1.1804	0.000612
RP11-146I2.1	25.26264927	5.133806	1.1786	0.000627
GS1-465N13.1	170.0663586	61.53982	1.178	1.85E-05
CARD9	1043.164219	348.952	1.1743	9.80E-05
CNGA1	231.8030513	78.53599	1.1704	9.19E-05
RP11-221N13.4	51.87843228	3.018444	1.1671	0.000401
IL11	451.225863	156.9067	1.1662	6.67E-05
ELFN2	195.4253959	47.73647	1.1642	0.000638
ZSCAN18	123.3641361	43.01197	1.1513	0.000104
MPP4	63.04338246	20.46986	1.1392	0.000291
NKX6-1	128.2179111	47.67968	1.1363	4.48E-05
MR1	393.9260186	137.0403	1.1273	0.000197
SLIT2	89.12595108	21.00806	1.1181	0.00115
FAM167B	66.37902476	21.92311	1.1093	0.000482
CNRIP1	64.52688469	18.42386	1.1047	0.000995
NTNG1	278.0206085	90.71195	1.1025	0.000593
PDGFB	362.8945479	141.0584	1.0964	6.52E-05
KCNAB2	391.4835991	129.8695	1.0962	0.0006
MAST1	421.198047	140.5281	1.0923	0.000619
TMEM156	412.0944561	173.2413	1.09	2.63E-06
PDZD7	57.19893315	16.39261	1.0854	0.001291
TRIM6	130.5595197	41.49449	1.0803	0.000975
NKX1-2	88.89761424	34.86025	1.0803	9.53E-05
UAP1L1	292.9173793	119.2103	1.0705	4.48E-05
SP5	88.14113798	33.00009	1.0655	0.000344
HERC5	173.1445	67.30219	1.0638	0.000203
SOX15	245.2163888	88.65073	1.059	0.000626
PLK3	549.1658677	211.6416	1.0543	0.000309
TMC6	1610.363728	619.2269	1.0393	0.000476
LHX1	1753.608156	713.5009	1.027	0.000248
IDUA	384.4937226	153.0715	1.0266	0.000397
C16orf45	58.9794532	22.48409	1.0248	0.000736
SAMD9L	163.9299264	66.50988	1.0231	0.000293
MAPK13	1412.981527	602.5067	1.0115	0.000127
PDP1	1341.046417	577.9918	0.99873	0.000155
WNK4	382.2935638	159.4488	0.99696	0.000402

HPCAL1	2906.691578	1285.424	0.9957	6.57E-05
CLDN4	9777.143135	4117.283	0.99239	0.000368
SPRY1	291.3398913	118.4316	0.9838	0.000892
RTEL1-TNFRSF6B	221.7756629	97.40368	0.98377	0.000162
STK32C	270.6696788	109.9122	0.98238	0.000918
STX1A	843.0095942	366.2303	0.98152	0.000247
HDAC9	1191.535005	536.7579	0.96855	0.000124
RP11-115C21.2	142.3059383	59.2272	0.95825	0.001223
SLC2A12	485.8525166	223.0074	0.94086	0.000234
USP18	178.643326	80.2205	0.93354	0.000573
CCDC149	165.9667553	74.29333	0.92123	0.001001
FAM89A	227.3285463	101.5821	0.91837	0.001117
IDS	1131.911016	535.3378	0.91653	0.000215
KLF2	119.812496	54.69074	0.9144	0.000822
MACC1	4859.715113	2431.763	0.90308	9.12E-06
CYR61	1359.720084	633.5014	0.89034	0.001048
FRMD5	546.7516928	276.3982	0.88617	1.91E-05
TMEM175	320.7445909	155.2113	0.88561	0.000417
CTBP1-AS1	517.2854456	252.6655	0.87701	0.000419
FAM84A	638.1366456	313.3314	0.86453	0.000614
ZDHHC2	446.0483827	218.3685	0.86118	0.000793
CNTN1	280.8744663	139.9023	0.85561	0.000461
EREG	2398.441646	1215.984	0.85245	0.000249
SOCS3	572.9092153	285.5217	0.8475	0.000746
OVOL1	287.4965312	149.1067	0.83964	0.000142
MFHAS1	2422.304932	1223.603	0.83344	0.000839
BMP4	813.3919787	413.8594	0.83285	0.000638
RIN3	781.0356069	394.7427	0.83215	0.000884
ABHD11	708.5184233	370.2599	0.82506	0.000225
GALK1	305.9648203	155.8562	0.82137	0.001074
C6orf120	381.1387399	201.0352	0.81334	0.000271
TRIM8	4080.080771	2142.23	0.80834	0.000515
PPP1R35	290.1448097	150.4463	0.80832	0.001009
WNT7A	10955.52941	6091.655	0.78341	1.65E-05
ZNF518B	478.5944238	258.2309	0.77013	0.001159
TSTD1	239.0508289	133.2847	0.74731	0.000621
SYNGR2	1154.585381	646.4938	0.74038	0.000806
SCD5	956.2043339	537.6231	0.73663	0.000806
ARHGAP29	2150.112753	1216.015	0.72978	0.000824
MAMDC4	613.2666866	347.7354	0.72324	0.001113
DOCK2	1826.687045	1081.17	0.69577	0.000205
SV2A	1009.223395	599.6179	0.68693	0.000367
SPSB1	370.9330119	218.8823	0.68385	0.001099
EVA1A	520.3461207	309.3965	0.67566	0.001058
RNF135	537.1909415	329.3851	0.64813	0.00063

BHLHE40	484.7117895	296.2538	0.64526	0.001256
SEMA7A	2293.275202	1414.269	0.63816	0.000933
ALCAM	4735.773254	2954.216	0.62934	0.000568
HEXDC	691.3824891	433.5209	0.62225	0.000735
THAP7	582.0473909	368.7404	0.6106	0.000656
CC2D1B	1378.951253	875.3713	0.60368	0.001268
ERCC3	1027.871182	1524.975	-0.53453	0.001176
ACTR3	2013.024302	3063.772	-0.56597	0.000979
RSF1	935.2669663	1425.261	-0.56776	0.000925
ATG13	697.3992881	1062.739	-0.56871	0.000776
HRAS	1310.720969	2016.072	-0.57795	0.001074
LRP11	453.012139	700.6581	-0.58294	0.001233
CERS6	815.8622613	1258.07	-0.5848	0.000543
IWS1	758.7837765	1173.667	-0.58708	0.000715
SORL1	2189.26744	3404.118	-0.58966	0.001132
MGAT5	1521.956666	2361.783	-0.59063	0.000668
PCNT	1535.140297	2388.585	-0.59279	0.000814
CSNK2A1	1482.908523	2299.396	-0.59328	0.000397
PTTG1IP	6264.556275	9756.836	-0.59837	0.000394
SEPT10	566.3828195	887.2776	-0.60357	0.000526
SPCS2	1013.032699	1590.023	-0.60492	0.000635
MZT2A	1151.496446	1823.74	-0.61347	0.000756
AMBRA1	686.7581543	1093.618	-0.61793	0.00087
MARCH7	654.0153392	1040.078	-0.62171	0.000516
UBE2G2	1452.770387	2310.514	-0.62712	0.000185
RCN1	1589.756271	2559.37	-0.63049	0.000871
FKBP9	1532.440005	2443.758	-0.63061	0.000188
U2AF1	2172.213493	3494.387	-0.63084	0.000809
BTBD10	455.1347601	733.4581	-0.6325	0.000872
ESF1	379.1539866	610.3643	-0.63649	0.00045
NCOA3	736.7117573	1198.2	-0.64067	0.001047
NAP1L4	2017.00274	3280.188	-0.64255	0.000821
FAR1	986.0295814	1592.615	-0.64905	9.77E-05
GNAQ	730.4061938	1196.902	-0.6504	0.000846
APMAP	1729.074282	2837.229	-0.65163	0.000891
MADD	906.4196845	1473.663	-0.65367	0.00016
AKAP13	957.3216403	1571.241	-0.65384	0.000683
CHID1	1277.157949	2091.131	-0.65567	0.000419
RALB	713.4597461	1163.892	-0.65864	0.000141
RTN3	2711.241714	4497.655	-0.66033	0.00116
SNN	745.1959513	1227.871	-0.66078	0.000477
CLNS1A	697.2676784	1150.306	-0.66262	0.000543
UNC45A	1066.180203	1747.17	-0.66276	0.000178
BAZ2B	527.0661881	876.2924	-0.6653	0.000845
PLEKHA7	828.2075608	1379.793	-0.66597	0.001031

COPB1	768.1900034	1275.463	-0.66626	0.000752
TANC1	781.8804428	1286.422	-0.66841	0.000131
SALL4	306.3692952	510.115	-0.66969	0.000707
DENND5A	546.4237463	907.8305	-0.67218	0.000383
SEMA4B	2367.554909	3900.862	-0.67734	3.42E-05
FAM111A	830.0749765	1395.913	-0.67833	0.000875
ARFIP2	769.9197457	1293.81	-0.67931	0.000662
ITSN1	621.7778176	1031.667	-0.67971	0.000124
PSPC1	577.2537252	975.367	-0.68193	0.000959
PRKRIR	496.6274467	831.3532	-0.68285	0.00033
BCL2L11	422.8365225	719.3652	-0.68589	0.001277
TALDO1	1828.049992	3083.26	-0.69529	0.000175
STIM1	954.7740518	1646.781	-0.69903	0.001226
CAPRIN1	3907.731953	6617.531	-0.70261	0.000121
ERMP1	1110.711958	1902.924	-0.71281	0.000167
BTBD1	590.7195617	1018.172	-0.71879	0.000214
ZBTB39	312.0543028	547.6028	-0.72046	0.001006
NUP98	1646.612911	2809.918	-0.72064	2.49E-05
FBXO22	394.1947692	688.4527	-0.72618	0.000435
FEN1	1515.046175	2623.805	-0.73252	6.15E-05
PTPN4	167.4497229	297.9031	-0.73347	0.00097
VIMP	248.0928098	443.4109	-0.73555	0.001219
RBMS1	794.3561595	1374.217	-0.73678	2.59E-05
PDXK	4082.21942	7330.428	-0.73804	0.001288
XPO4	351.8453679	629.8633	-0.73838	0.000978
DHCR7	1656.226133	2956.22	-0.7454	0.000519
UBXN4	1039.975554	1839.867	-0.74629	0.000196
FMNL2	1426.185573	2500.227	-0.74811	4.26E-05
AMMECR1L	572.0878683	1009.489	-0.75776	3.47E-05
SIRT3	186.8873841	339.6408	-0.75941	0.000635
RHOG	420.2878648	746.0405	-0.76011	5.84E-05
TBC1D2B	446.5387262	795.0673	-0.76326	7.18E-05
RAB6A	570.117639	1010.107	-0.76436	2.08E-05
EFTUD1	211.663183	386.9494	-0.76817	0.000664
HS6ST1	1354.203653	2470.287	-0.77705	0.000226
IGSF3	787.7142963	1421.852	-0.77788	6.69E-05
WHAMM	275.2514812	500.0287	-0.78092	0.000107
FASTKD5	146.6298072	268.9422	-0.7856	0.000183
SMARCA2	560.0558663	1039.347	-0.79931	0.000146
BACE2	503.3804201	927.0446	-0.80014	6.65E-05
ZMYND8	1209.178135	2295.07	-0.80166	0.000616
MALL	373.8522461	709.0691	-0.80349	0.000499
PARVA	570.7914915	1056.848	-0.80613	6.29E-05
TNKS1BP1	2694.823735	5041.892	-0.81122	0.000106
KLHL25	261.8224111	502.0508	-0.81926	0.000436

INTS9	158.2667611	301.4427	-0.82088	0.000255
SMOX	362.2529756	693.8368	-0.82751	0.000221
TTC21B	113.6231498	218.7198	-0.82753	0.000336
OS9	1202.155307	2237.273	-0.83068	4.01E-06
ARL14EP	138.3526279	266.9804	-0.83701	0.00018
DNAJA4	919.8876372	1747.008	-0.83741	3.63E-05
PSAT1	274.0246969	539.2919	-0.84094	0.000449
KLHL9	402.6785681	812.5321	-0.84135	0.001203
MRPS6	203.0381282	390.1519	-0.84349	6.97E-05
SNX33	548.9297686	1062.038	-0.84394	0.000118
GLUL	6181.953803	12156.25	-0.84558	0.000317
EPPK1	3238.945564	6305.403	-0.84834	0.000135
RAB4A	140.8807044	273.5906	-0.84901	9.18E-05
FAM111B	517.5774137	1013.282	-0.86007	9.01E-05
DDX58	253.7233342	496.0623	-0.86232	6.23E-05
KCNJ15	678.6767416	1407.857	-0.86378	0.00114
BX322557.10	91.22181791	182.9468	-0.86548	0.000277
AC103965.1	334.8468523	656.9821	-0.86933	5.04E-05
CNTNAP2	456.0583279	930.8912	-0.86945	0.000537
OAS3	1683.598891	3368.691	-0.87571	0.000124
DDB2	258.2902867	521.06	-0.87961	0.000167
ZSCAN2	192.1134822	388.529	-0.88257	0.000166
RP11-658F2.8	62.60789347	130.2487	-0.88363	0.000633
SRGAP3	272.6010866	569.7706	-0.88779	0.000555
EIF3F	1859.803852	3794.105	-0.89215	0.000142
CTNND1	3844.864213	7473.374	-0.89395	2.56E-07
RRP8	199.5160869	408.0508	-0.90153	0.000102
UBE2L6	132.0714719	275.0296	-0.90451	0.000228
DTX4	1221.233198	2546.048	-0.91133	0.00014
HNRNPUL2	278.5012459	571.1221	-0.91141	4.76E-05
GPCPD1	91.2066652	198.6586	-0.91741	0.000653
SERPINH1	3204.750194	6459.615	-0.9287	9.37E-07
B4GALT1	1203.807874	2579.13	-0.93035	0.00019
MDK	4930.17889	11003.69	-0.93116	0.000684
ZNF618	805.977903	1748.536	-0.93724	0.000225
PPARGC1B	144.6582624	311.5261	-0.93867	0.000156
CKB	1773.328051	3641.041	-0.94338	2.00E-06
AP001053.11	149.8277447	324.2799	-0.94401	0.000135
FRMD3	181.0455022	392.0345	-0.94636	0.000125
BAG1	469.2288899	1118.739	-0.95322	0.001233
SLC52A3	116.9735244	250.7105	-0.95889	2.58E-05
GAB2	175.104197	405.1625	-0.95958	0.000565
BDNF	108.2771124	237.5322	-0.96092	0.000118
CST3	975.9804959	1993.151	-0.96129	2.54E-08
LSS	972.0429781	2170.859	-0.96368	0.000194

AP006621.5	28.50848572	70.03478	-0.96825	0.001207
ABCC4	183.5933481	394.3554	-0.9687	2.23E-05
HMGCR	987.2775133	2347.462	-0.9719	0.000714
DAPK1	482.1309511	1054.703	-0.9749	3.69E-05
FADS1	1648.60039	3476.177	-0.98282	4.32E-07
RAB20	77.84451508	171.589	-0.98628	3.12E-05
CYP27C1	154.8246221	397.3793	-1.0048	0.000934
ST5	519.643836	1104.955	-1.006	3.58E-08
AP006621.1	20.19522649	53.88827	-1.0065	0.00116
SERINC5	112.3239593	261.0129	-1.0122	8.96E-05
CAND2	249.397367	632.9927	-1.0124	0.000674
CIB2	61.01937287	155.021	-1.0168	0.000636
HAPLN3	45.70336923	111.1013	-1.0169	0.000243
PTPRJ	157.513327	356.9473	-1.0209	1.34E-05
HMBOX1	82.62170728	189.3719	-1.0219	2.27E-05
SWAP70	673.7332386	1495.374	-1.0404	3.03E-07
NRG4	33.89926023	91.51239	-1.0461	0.000689
EPHB3	52.32925345	151.5888	-1.0496	0.0011
NMB	161.8379478	385.6237	-1.052	3.19E-05
ZNF385A	187.0744352	491.0614	-1.0649	0.000267
MYCL1	30.88370471	82.06424	-1.0775	0.00023
ALX3	1.862509466	18.38353	-1.079	0.001239
FADS2	2216.114479	5618.943	-1.0846	6.67E-05
ATF7IP2	1.821165988	15.37073	-1.0935	0.001257
SLC14A1	2.579789316	35.31779	-1.1039	0.000822
SPINK5	40.0361416	111.8856	-1.11	0.000191
SCPEP1	928.5583263	2174.977	-1.1133	3.07E-08
RP11-551L14.1	51.43366291	137.8787	-1.1249	5.31E-05
CDON	80.44876845	211.7695	-1.1259	3.71E-05
HAP1	211.7375445	528.0398	-1.1316	2.72E-06
BCAM	800.6572929	2325.847	-1.1327	0.000196
LGR6	8.621398426	50.19517	-1.1337	0.001006
VANGL2	457.1369638	1094.915	-1.136	4.08E-08
PTN	2.196156319	22.9387	-1.1417	0.000697
ZNF542	12.80637039	55.53761	-1.1454	0.000849
CDKN1C	211.4085976	627.9377	-1.1477	0.000177
MGAT3	5.480160012	37.48779	-1.1624	0.000723
WNT5A	202.5234457	546.8224	-1.1678	1.29E-05
PRKCDBP	199.196446	570.2679	-1.1742	4.71E-05
SYNGR1	31.14718106	153.2656	-1.1772	0.000647
AC112229.1	13.09764459	45.43307	-1.1825	0.00029
SYT8	53.04334263	222.1212	-1.1878	0.00049
TFF2	0.837713708	29.31333	-1.1909	0.000248
ACSS1	108.3909764	292.5003	-1.1953	3.17E-06
ZNF334	0	14.56257	-1.2108	0.000163

RNF24	128.711074	332.0525	-1.2111	3.16E-08
SOSTDC1	1.174957137	27.0803	-1.2193	0.000216
LYPD6B	70.82119485	215.5137	-1.2207	3.56E-05
CD37	181.9902382	538.3528	-1.2236	1.77E-05
SYNPO	50.76367312	167.4742	-1.2247	9.21E-05
H19	0	3892.417	-1.2259	0.000105
RHOU	27.58911424	124.6609	-1.2262	0.000329
CHGB	10.25527941	56.99866	-1.2268	0.000382
SYT7	71.71945261	263.0501	-1.2271	0.000182
PPP2R2C	0.558475806	18.65628	-1.235	0.000175
MRAS	8.980349992	35.11997	-1.2453	0.000172
ALPP	0.670890282	40.25849	-1.2483	0.000118
TMEM151B	29.46261638	139.1984	-1.2484	0.000264
AP3B2	45.89916821	157.6261	-1.2509	7.71E-05
FLRT3	272.6221826	896.96	-1.2524	4.36E-05
RLTPR	42.2736414	168.0897	-1.2544	0.000159
KCTD14	0	16.44607	-1.2583	9.94E-05
FAAH	160.7986082	513.435	-1.2589	2.37E-05
MX1	30.52599971	93.26227	-1.2591	7.99E-06
SLC6A16	43.88456769	144.5562	-1.2668	3.12E-05
CTD-2532K18.2	1.50860399	21.07173	-1.2684	0.000179
BEND7	1.525266038	22.87913	-1.2703	0.000168
EDIL3	11.03796089	96.95555	-1.2729	0.000205
KRT14	71.40301683	247.4435	-1.2762	4.26E-05
IFITM3	238.0366199	652.886	-1.2833	9.98E-09
UPK3B	200.3287987	686.6727	-1.2882	2.85E-05
IFITM2	17.79747313	67.38461	-1.2896	7.13E-05
TGM2	4240.588899	11284.71	-1.2904	2.78E-11
GJB6	93.68507271	420.9447	-1.3209	8.37E-05
ISG20	23.48724956	79.55101	-1.3353	5.00E-06
ZNF114	5.155155824	31.78781	-1.3353	0.000106
CASP1	6.897231432	62.31832	-1.3359	0.000104
CRTAC1	3.284003693	34.73698	-1.3422	9.24E-05
NSG1	123.7931348	462.8868	-1.3507	1.61E-05
RP11-34A14.3	5.28001249	49.80032	-1.3568	8.14E-05
B3GALT5	29.59295391	112.8103	-1.3585	1.41E-05
VGLL1	21.02893772	132.6802	-1.3653	7.47E-05
RIMKLB	215.9046367	661.0874	-1.3703	3.07E-08
CD81	730.1409149	2344.553	-1.3909	1.06E-07
VWA2	57.4791458	237.3524	-1.3948	1.43E-05
ITGB6	716.0540922	2307.714	-1.3964	9.05E-08
ADAM23	3.780051165	41.12569	-1.3984	4.83E-05
METTL7A	6.458566202	61.84792	-1.4086	4.39E-05
DMBT1	1.133613659	41.01423	-1.4116	2.41E-05
UNC5A	35.8228772	152.9336	-1.413	1.11E-05

GRID1	9.916789421	85.29139	-1.4134	4.25E-05
SIDT1	21.49483734	97.2321	-1.437	1.00E-05
WNT9A	29.96060797	156.8807	-1.4625	1.30E-05
MAF	1.062542661	25.96294	-1.4672	1.54E-05
PADI3	7.830074285	40.34301	-1.4703	1.01E-05
LSP1	20.24376312	87.07247	-1.4759	2.97E-06
RGMA	65.40139038	275.9489	-1.4998	1.24E-06
TNNI2	9.138530753	91.0414	-1.513	1.19E-05
RHCG	0.854375756	21.84642	-1.538	6.89E-06
ZNF502	0.575137853	20.41078	-1.5667	4.40E-06
CPA4	129.0271492	638.1126	-1.5714	9.69E-07
EDAR	63.80393546	254.6595	-1.5799	1.77E-08
KRT17	717.3168154	3394.786	-1.5913	3.57E-07
SPOCK2	76.77486797	484.1676	-1.6024	1.90E-06
FGD2	20.12415549	87.80733	-1.6205	2.35E-08
ALPL	5.00879397	47.40867	-1.6586	1.44E-06
TSPAN11	3.091875553	91.53536	-1.689	7.88E-07
CPAMD8	10.42147956	117.8324	-1.7121	7.02E-07
GCA	14.05724167	95.84538	-1.7463	1.13E-07
MX2	18.25957323	127.0371	-1.7671	9.66E-08
SDR42E1	0.279237903	47.90985	-1.7914	1.39E-07
ITGB7	12.75175849	134.3358	-1.8372	7.90E-08
NYNRIN	15.09489995	138.5664	-1.8515	4.29E-08
C21orf88	6.334535768	53.3335	-1.8527	2.60E-08
KRT4	0.670890282	133.2486	-1.8911	2.73E-08
KIAA1211L	47.32212638	331.8019	-1.9239	1.61E-09
TMPRSS3	1.174957137	60.97493	-2.0097	5.56E-09
CCAT1	1.166937754	73.55967	-2.0682	2.01E-09
LGALS9	7.59217986	97.03326	-2.1753	8.76E-11
KCNG1	5.671664869	61.86946	-2.2054	1.07E-11
IGFBP3	804.1014766	4965.612	-2.2594	3.19E-21
OAS1	43.70840405	517.6777	-2.2899	2.65E-12
TP53I11	2.420985272	52.1696	-2.3502	4.16E-12
TMTC1	0.950128184	61.08514	-2.3771	5.61E-12
PELI2	13.32986964	264.5614	-2.3958	1.67E-12
PADI2	97.4763682	1257.254	-2.5304	1.57E-15
TNNT3	5.146513159	247.8481	-2.8895	1.77E-17
ANXA6	8.50898395	378.3228	-3.1553	2.81E-21
ADH1C	0.279237903	102.2976	-3.4926	1.11E-25

Table S6: OVOL1 and OVOL2 targets

OVOL1 Targets (563)	OVOL1 and OVOL2 overlapping targets (271)	OVOL2 Targets (293)
AASS	A2ML1	ANKRD26P1
ABHD4	AGR2	CH17
ABI3BP	AIM1L	CTBP2P1
ACBD4	ALDH3B2	KIF16B
ACER2	AMN	MT
ACPP	ANKRD18A	RNU4
ACTRT1	ANO9	RNU6
ADAMTSL1	AP1M2	SVEP1
ADGRF1	ARHGAP40	ABCC12
ADH7	ATOH1	ABHD11
AICDA	ATP2C2	ACSF2
AIM1	ATP6V0A4	ACY3
AK3	B3GNT3	ADGRF2
AKR1B15	B4GALNT3	ADGRG7
ALKBH8	BAIAP2L1	ADGRV1
ALOX12B	BARX2	AGR3
AMER1	BCL2L10	ALOXE3
ANKRD22	BICDL2	ANKRD30A
ANKRD36	BIK	ANXA8
ANXA8L1	BNIPL	APOA5
AQP3	BSPRY	ARHGAP8
AQP5	C10orf99	ARHGEF16
ARAF	C19orf33	ARHGEF28
ARGFX	C1orf106	ARHGEF38
ARHGAP15	C1orf116	ARL14
ARHGAP19	C1orf210	ARRDC1
ARHGAP27	C2orf54	ASCC2
ARHGAP44	C6orf132	ATP13A4
ARHGAP6	CALML3	ATP13A5
ARHGEF35	CALML5	AWAT2
ARID1A	CAMSAP3	BPIFA3
ARL2	CBLC	BPIFB1
ARNTL	CCDC120	C2orf72
ASB15	CCDC187	C6orf222
ASCC1	CCNO	C9orf152
ATM	CD164L2	CAPN13
ATP10D	CD24	CAPNS2
ATP12A	CDC42BPG	CAPZA3
ATP1A3	CDH1	CASR
ATP2A1	CDS1	CCDC185
ATP2A2	CEACAM5	CCDC88C

ATP6AP1L
B3GALT5
B3GNTL1
BCL2L14
BCL3
BDH1
BEND2
BEND7
BFSP1
BLNK
BORCS8-MEF2B
BRCA2
BRD2
BRWD1
C11orf58
C12orf4
C12orf56
C15orf48
C16orf71
C1orf112
C1orf68
C20orf202
C21orf2
C4orf26
C8orf82
CA12
CAB39L
CABLES1
CAMK2A
CARD8
CASP14
CASZ1
CBX1
CCDC103
CCDC112
CCDC129
CCDC155
CCDC77
CCDC80
CCDC81
CCDC88A
CCNL2
CCT5
CCT7
CD1A
CEACAM7
CGN
CKMT1B
CLDN4
CLDN7
CNFN
CNKSR1
COL17A1
CRB3
CRCT1
CWH43
CXADR
CXCL17
CYP2C18
CYP2C9
CYP2J2
DCDC2
DEF6
DEFB1
DEGS2
DMKN
DSG3
DSP
EDARADD
EFNA3
EHF
ELF3
ELMO3
EPCAM
EPHA1
EPN3
EPS8L1
EPS8L2
ERBB2
ESPN
ESRP1
ESRP2
EVPL
EXPH5
F2RL1
FA2H
FAM110A
FAM25A
FAM3B
FAM3D
CDHR1
CHDH
CHMP4C
CHRM1
CKMT1A
CLDN3
CLDN8
CNNM4
COL6A5
CPTP
CSN3
CSPG4
CYB561
CYLC1
CYP24A1
CYP2S1
CYP3A5
CYP4F11
CYP4F2
DDR1
DGKK
DMBT1
DOK7
DOLPP1
DQX1
DUOX1
ELF5
ENTPD8
EPB41L4B
EPHA10
ERBB3
ERN2
FAAH
FAAH2
FAM109A
FAM71B
FAM71E2
FAM84A
FDCSP
FETUB
FOXA2
FOXA3
FOXJ1
FOXQ1
FRAT1

CD33
CDCP1
CEBPA
CELSR1
CEP162
CEP170
CEP89
CERS3
CFHR5
CHP2
CHRM3
CHST10
CIB1
CKLF
CKLF-CMTM1
CLCA2
CLCA4
CLCN6
CLDN12
CLDN23
CLIC3
CLINT1
CLP1
CNOT2
CNOT6L
COCH
COL5A2
COQ10B
CORO2A
CPNE4
CPNE6
CRISP1
CRLF2
CRNN
CS
CST6
CSTA
CTH
CTNNA2
CTSV
CUBN
CUL2
CWC27
CYLD
CYP27B1
FAM83B
FAM83E
FAM83F
FAM83H
FAM83H-AS1
FANK1
FERMT2
FOLR1
FOXA1
FOXN1
FUT3
FXYD3
GALNT3
GGT6
GJB5
GPR87
GPRIN2
GPX2
GRB7
GRHL2
HMGCS2
HNF1B
HNF4G
HSD11B2
IGFL1
IGSF9
IL1A
IL36G
ILDR1
IRF6
ISX
JUP
KDF1
KLC3
KLF5
KLK11
KLK12
KLK7
KLK8
KRT14
KRT15
KRT17
KRT19
KRT23
KRT5
FRAT2
FRMPD2
FSHB
FUT2
GJB1
GMNN
GRAMD1C
GRAMD2
GRID2IP
GSTA1
GSTA3
GSTO2
GUCA2B
HEPACAM2
HID1
HOOK2
HOXB13
HOXC13
HS3ST6
HSBP1L1
HSD17B2
IFNLR1
IHH
IL36A
IRX2
IRX3
IRX4
ITGB6
KCNK10
KIAA1324
KIAA1522
KIF12
KLHDC7A
KLK10
KLRG2
KRT18
KRT32
KRT33A
KRT74
KRT83
KRTAP11-1
KRTAP3-2
KRTAP4-7
KRTAP4-8
KRTAP9-3

CYP2A7
CYP4F22
CYSRT1
DAG1
DAPL1
DDHD2
DDI2
DEFA5
DENND1C
DERL3
DGKH
DLGAP5
DLX3
DNAJC13
DNHD1
DNTTIP1
DOCK8
DOLK
DSC1
DSG1
DSTN
DTNB
DUSP14
EFCAB5
EFNA4
EML2
EPB41L3
EPHA2
EPHX3
EPN2
ESPL1
ESR2
ESYT2
EVC
EXOC3L2
EXOC6
FAM110C
FAM149B1
FAM177B
FAM83A
FAM83C
FAM83G
FAM96A
FAT2
FBLIM1
KRT6B
KRT6C
KRT75
KRT78
KRT80
KRTCAP3
LAD1
LAMB3
LARGE2
LEMD1
LGALS7
LINC00675
LIPH
LIPM
LLGL2
LRRC31
LY6D
LYPD6B
MACC1
MAL2
MAP3K9
MAP7
MAPK13
MARVELD2
MARVELD3
MFSD6L
MISP3
MNX1
MROH2A
MST1R
MT4
MUC15
MYCL
MYH14
MYO5B
NCCRP1
NECTIN4
NIPAL1
NKPD1
OCLN
OSBP2
OTOP2
OTOP3
PADI3
PDZK1IP1
LCE1D
LCE2A
LCE3D
LCE6A
LENG9
LFNG
LIMK2
LMNTD2
LMTK3
LPAR2
LRRC26
LSR
LYPD8
MAGIX
MKRN2OS
MLPH
MS4A12
MS4A8
MSMB
MYO3A
NOTUM
NPM2
NR0B2
NTF4
NUP62CL
NUPR2
OTOS
OTX1
OVOL1
PAK4
PAX2
PCP2
PDCL2
PDZD3
PGAP2
PGLYRP3
PIGR
PIK3C2G
PLCH1
PLEKHS1
PLPP2
PNLIPRP3
PPP1R13B
PRDX5
PRKCZ

FBXL16
FBXL2
FBXW12
FGFBP1
FGFR3
FGL1
FHDC1
FOXB1
FOXJ2
FPR1
FUT6
GALE
GATB
GCH1
GCNT3
GJB2
GJB3
GJB6
GJC1
GK2
GKAP1
GLG1
GLT8D1
GLTP
GNGT1
GNL2
GNMT
GOLGA2
GOLT1A
GON4L
GPAT2
GPBP1
GPR143
GPR85
GRHL1
GRHL3
GYG1
GYS2
HDAC7
HEPH
HERC5
HES2
HIBADH
HIC1
HMGA1
PGLYRP4
PKP3
PLA2G3
PLA2G4D
PLA2G4F
PLEK2
PLEKHG6
PLS1
POF1B
PPP1R13L
PPP1R14C
PROM2
PRR15L
PRRG2
PRSS16
PRSS22
PRSS8
PTK6
RAB11FIP4
RAB25
RAET1L
RASAL1
RASSF7
RBBP8NL
RBM47
REEP4
RHOV
RNF128
RNF223
S100A14
S100A7
SAPCD2
SBSN
SCEL
SCNN1G
SDC1
SDCBP2
SERINC2
SERPINB12
SERPINB13
SERPINB4
SERPINB5
SFN
SH2D3A
SH3RF2
PRR15
PRR36
PRRG4
PSCA
PSD4
PSORS1C2
PTH
PTPN3
PWWP2B
RAB17
RAB27B
RASSF10
RASSF6
RDH13
RIPK4
RNF39
RNF43
RORC
RPL10L
RPS6KA1
RPTN
RXFP1
SAA2-SAA4
SBK1
SCGB1D2
SCGB2A2
SCNN1A
SDR16C5
SEMA4G
SERPINA11
SERPINA6
SERPINB11
SH2D4A
SH3YL1
SHD
SHH
SIPA1L3
SLC13A2
SLC22A23
SLC22A6
SLC29A2
SLC34A2
SLC37A1
SLC44A4
SLC5A8

HMGCL	SLC12A8	SLC6A14
HN1	SLC25A10	SLC6A19
HNF4A	SLC27A2	SMCO4
HNRNPA1L2	SLC5A1	SMIM6
HNRNPL	SLC9A3R1	SMPD2
HNRNPM	SLURP1	SMPD3
HOOK1	SMIM22	SMR3B
IDE	SMPDL3B	SOX9
IFFO1	SOWAHB	SPATA3
IFIT5	SOX21	SPINK4
IL11RA	SPACA4	SPRR2B
IL1RN	SPDEF	SPRR2F
IL22RA1	SPINK5	SPRR4
INCENP	SPINT1	SPTSSB
ING4	SPINT2	SPZ1
INTS6L	SPRR1A	SSTR3
IRX5	SPRR1B	STARD10
ITGA9	SPRR2E	STATH
IVL	ST14	STRBP
JAZF1	STAP2	STX19
KCND2	STXBP2	STYK1
KDELC2	SULT2B1	SULT1E1
KDM4D	SYT8	SYCE3
KIAA0368	SYTL1	SYNGR4
KIAA1211	TACSTD2	SYTL5
KIF2A	TGFA	TBATA
KIF3A	TGM3	TCERG1L
KIF6	TJP3	TCN1
KLHL2	TLCD1	TESMIN
KLK1	TMC4	TEX11
KLK13	TMC5	TEX33
KLK5	TMEM125	TEX35
KPRP	TMEM171	TFAP2C
KRT16	TMEM184A	TFCP2L1
KRT6A	TMEM45B	TFF1
KRTAP10-6	TMPRSS11E	THEG
KRTAP21-3	TMPRSS13	TLX1
KRTDAP	TMPRSS2	TM4SF5
KTN1	TMPRSS4	TMEM102
LAS1L	TNS4	TMEM139
LATS2	TPRN	TMEM238
LCE1A	TTC22	TMEM61
LCE1E	TTC39A	TMPRSS11F
LCE2B	UGT1A7	TMPRSS3
LCE5A	UNC93A	TNFRSF13B

LEPROTL1
LGALS7B
LGALS9C
LHX8
LIMA1
LIMS1
LIMS3
LIPI
LMX1A
LONRF1
LOR
LRFN5
LRP4
LRRC1
LRRC2
LRRC72
LRTM1
LTB4R
LY6G6C
LYPD3
LYPLAL1
LYST
MACROD2
MAGED1
MALL
MAP1LC3B2
MATN4
MC3R
MEP1A
MOGAT3
MPLKIP
MPZL3
MREG
MRPL15
MRPS31
MTHFR
MTPAP
MTRNR2L3
MUC21
MYRIP
N4BP3
NCALD
NCKAP5L
NCOA3
NCOA5
USP43
VAMP8
VAV3
WWC1
ZNF296
TNK1
TOX3
TRIM31
TRPV4
TRPV6
TSPAN1
TSTD1
TTC6
UGT2A3
UGT2B15
UGT2B17
UGT2B7
UGT3A1
UGT8
UNCX
USH1G
VIPR1
VSIG2
VTCN1
WDR34
WDR72
WFDC2
WNT4
WNT7A
XDH
YBX2
ZG16B

NCOA6		
NDUFAF2		
NELL2		
NFAM1		
NFATC3		
NFE2L3		
NGRN		
NIPAL4		
NISCH		
NLRP2		
NME9		
NMU		
NOP16		
NOX5		
NPHP4		
NPRL3		
NT5C1B		
NT5C1B-RDH14		
NUAK2		
NUP188		
NUP54		
NXN		
OR10A7		
OR1B1		
OR3A3		
OR4D9		
OR52B6		
OR52N2		
OR52R1		
OR6C70		
OR7C2		
OR8K1		
OVOL2		
OXNAD1		
P2RY2		
P2RY8		
PABPC5		
PADI1		
PAK6		
PAMR1		
PAQR5		
PARG		
PATE3		
PCDHA4		
PCM1		

PCNX1		
PCSK9		
PDE1A		
PDE3B		
PDLIM5		
PER2		
PFKFB4		
PHLDA2		
PHLDB3		
PI3		
PIP4K2B		
PITX1		
PKD1L1		
PKD2		
PKLR		
PKP1		
PKP4		
PLAC8		
PLAC9		
PLEKHN1		
PLS3		
PLXNB2		
POLK		
POLR2B		
POLRMT		
POR		
POU2F3		
PPARG		
PPP2R1B		
PPP2R2C		
PRADC1		
PRKCQ		
PRLR		
PRMT3		
PRRT3		
PRSS3		
PSMA1		
PSMD2		
PTGES3L-AARSD1		
PTPRD		
PTTG1		
RAB26		
RAB31		
RAB33A		
RABEPK		

RAD51AP1		
RAD52		
RAP1GAP		
RAPGEFL1		
RASGRF1		
RBM26		
RBM4		
RDH12		
RDH8		
RERE		
RGL4		
RHCG		
RNASEH2B		
RNF222		
RNF40		
RNF8		
RPL14		
RPL7L1		
RTTN		
RUNDC1		
RUSC1		
S100A16		
SAMD10		
SAMD9L		
SAMSN1		
SAP25		
SCMH1		
SCN8A		
SDK1		
SEMA6D		
SENP6		
SERPINB2		
SERPINB3		
SERTAD1		
SH3BP2		
SH3KBP1		
SHF		
SHQ1		
SIAH2		
SIGLEC8		
SIRT3		
SLAMF6		
SLC10A6		
SLC12A6		
SLC22A10		

SLC24A5		
SLC35C2		
SLC39A12		
SLC39A2		
SLC6A4		
SLC7A2		
SLIRP		
SLIT1		
SMURF2		
SNAI2		
SORBS1		
SOSTDC1		
SOX15		
SOX6		
SP6		
SP7		
SP8		
SPACA5		
SPAG5		
SPAG6		
SPDYA		
SPHK2		
SPINK7		
SPOCK2		
SPRR2A		
SPRR2D		
SPRR3		
SPTBN2		
SREBF1		
SSSCA1		
SSX2IP		
ST13		
ST18		
STON1		
SULT4A1		
SYDE1		
TAF15		
TANK		
TCEANC		
TECR		
TEX101		
TFEC		
TFG		
TG		
THAP1		

THAP8		
TIE1		
TLN1		
TMEM40		
TMEM63C		
TMPRSS11D		
TMSB4X		
TP63		
TPTE		
TRABD		
TRAF4		
TRAPPC9		
TRGC1		
TRIM16		
TRIM16L		
TRIM29		
TRIM36		
TRIM55		
TRO		
TRPV1		
TTC19		
TTC27		
TTN		
TTYH1		
TUBGCP5		
TUFT1		
TXNDC17		
TXNRD1		
UBQLN2		
UBR4		
UBXN4		
UBXN7		
UGT2A1		
UPRT		
USP33		
USP46		
USP47		
VAV1		
VCL		
VGLL4		
WDR27		
WDR35		
WDR62		
WFDC3		
WNK4		

WNT7B		
WRB		
XIRP2		
XK		
YTHDF1		
YWHAE		
ZBED2		
ZBTB7C		
ZDHHC15		
ZFP36		
ZKSCAN1		
ZMYND11		
ZMYND8		
ZNF165		
ZNF182		
ZNF2		
ZNF235		
ZNF48		
ZNF568		
ZNF577		
ZNF614		
ZNF717		
ZNF750		
ZNF76		
ZNF764		
ZNF85		
ZZZ3		
