# Supplementary material for: Targeting ketone body metabolism in mitigating gemcitabine resistance
Source: JCI Insight. 2024 Dec 20;9(24):e177840. doi: 10.1172/jci.insight.177840 (PMC11665555; doi:10.1172/jci.insight.177840)
Supplement: Unedited blot and gel images [file jciinsight-9-177840-s044.pdf]

Title: Targeting Ketone Body Metabolism in Mitigating Gemcitabine Resistance

Unedited Western Blot Images

Antibodies

OXCT1 (HPA061425, Sigma Aldrich), HMGCS2 (Ref, 20940S, Cell Signaling), CPT1A (Ref 12252S, Cell Signaling), PPARG (Ref sc-7273, Santa Cruz) OVOL1 (Ref PA5-41480, ThermoFisher), and OVOL2 (Ref NB030227, Novus Biological) β-Actin (Ref sc-47778, Santa Cruz) Laminb1 (Ref sc-377000, Santa Cruz) and Tom20 (Ref sc-17764, Santa Cruz).

1) Full unedited gels for Figure 2B:

Lanes 1: MW 2: RT4 3: UMUC3 4: 5637 5:T24

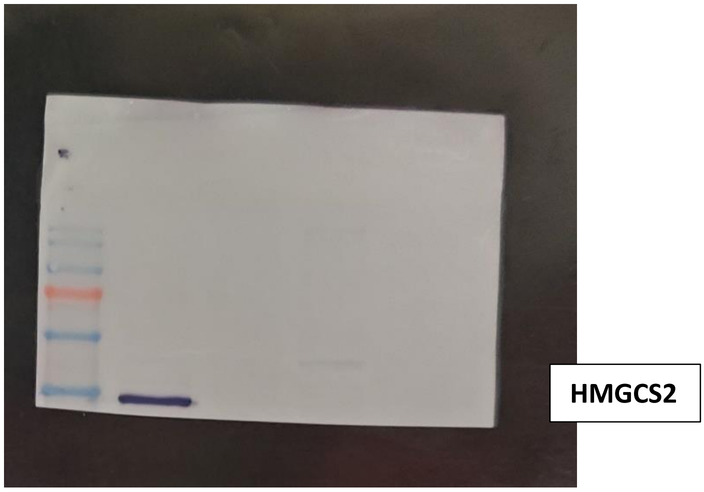

HMGCS2

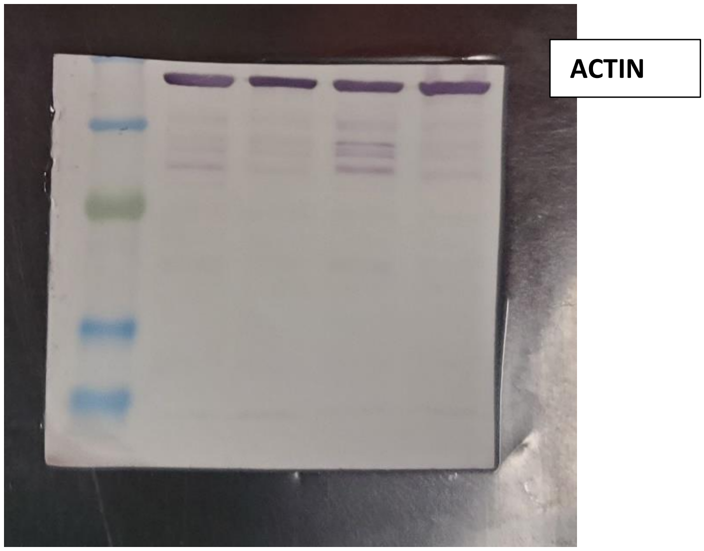

ACTIN

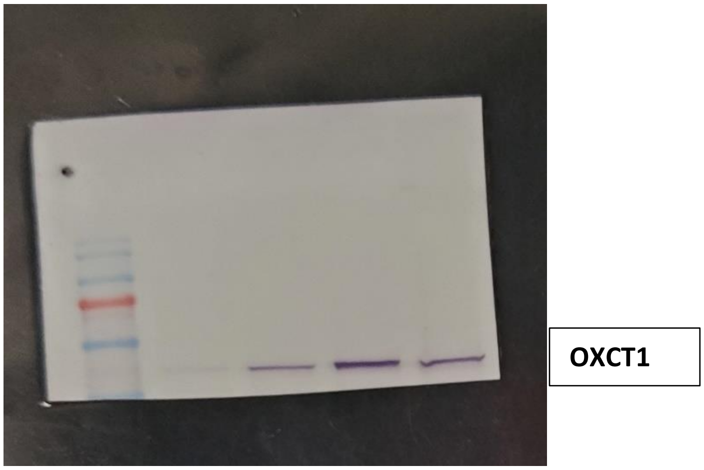

2) Full unedited gels for Figure 2E:

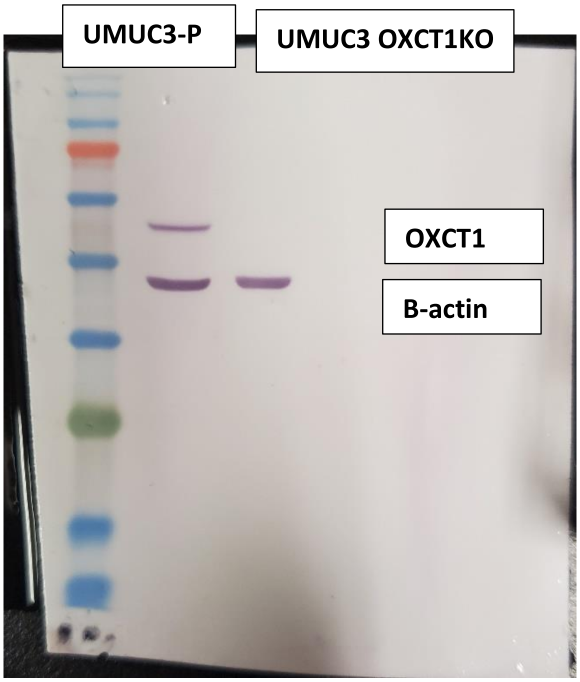

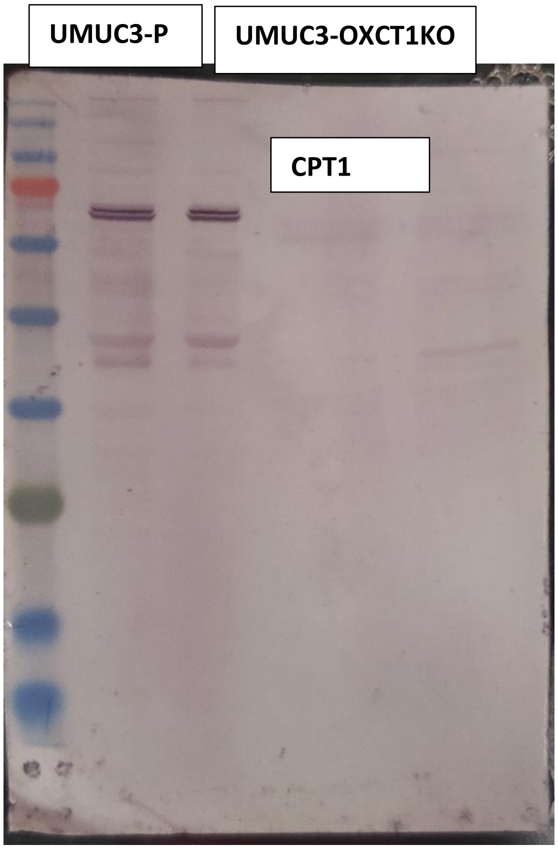
UMUC3-P
UMUC3-OXCT1KO
CPT1

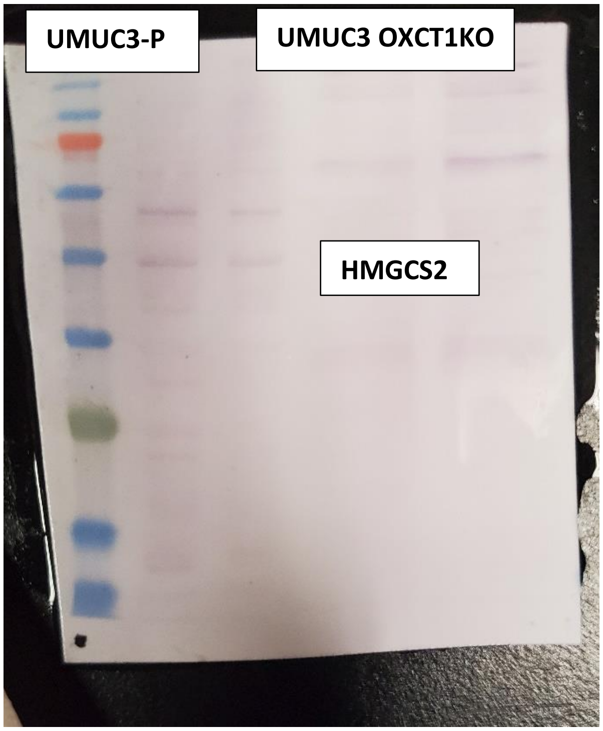
UMUC3-P
UMUC3 OXCT1KO
HMGCS2

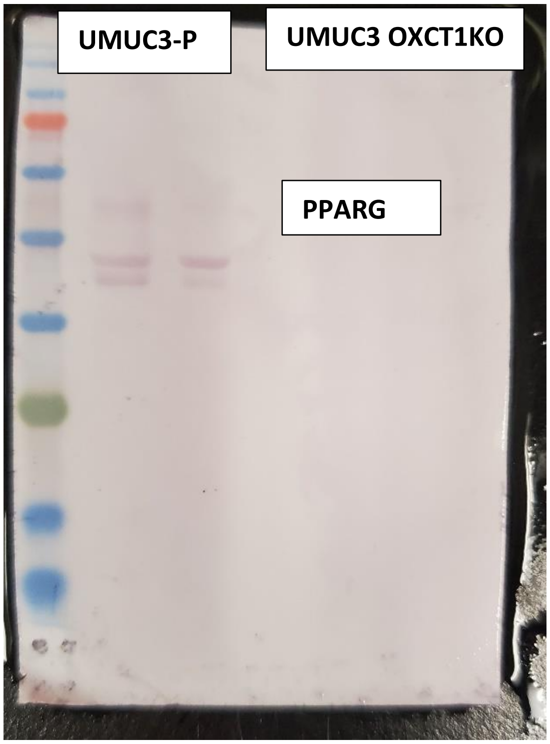

3) Full unedited images for figure 5

For all following images the lanes are:

L Cyt: Ladder Cytoplasmic Fraction.

L Nuc: Ladder Nuclear Fraction

1) 5637 Parental Vehicle

2) 5637 Parental Gemcitabine

3) 5637 GR Vehicle

4) 5637 GR Gemcitabine

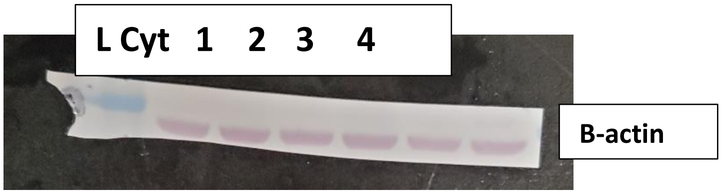

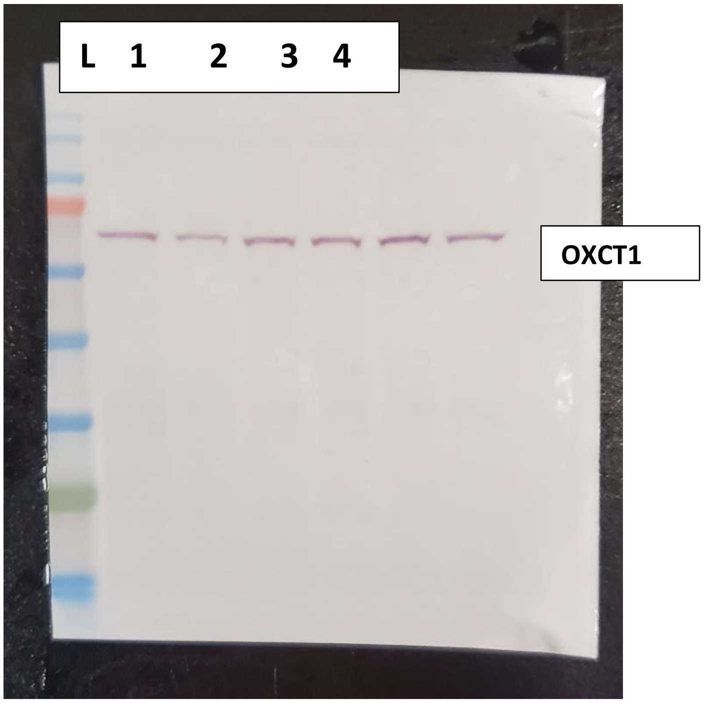
L 1 2 3 4
OXCT1

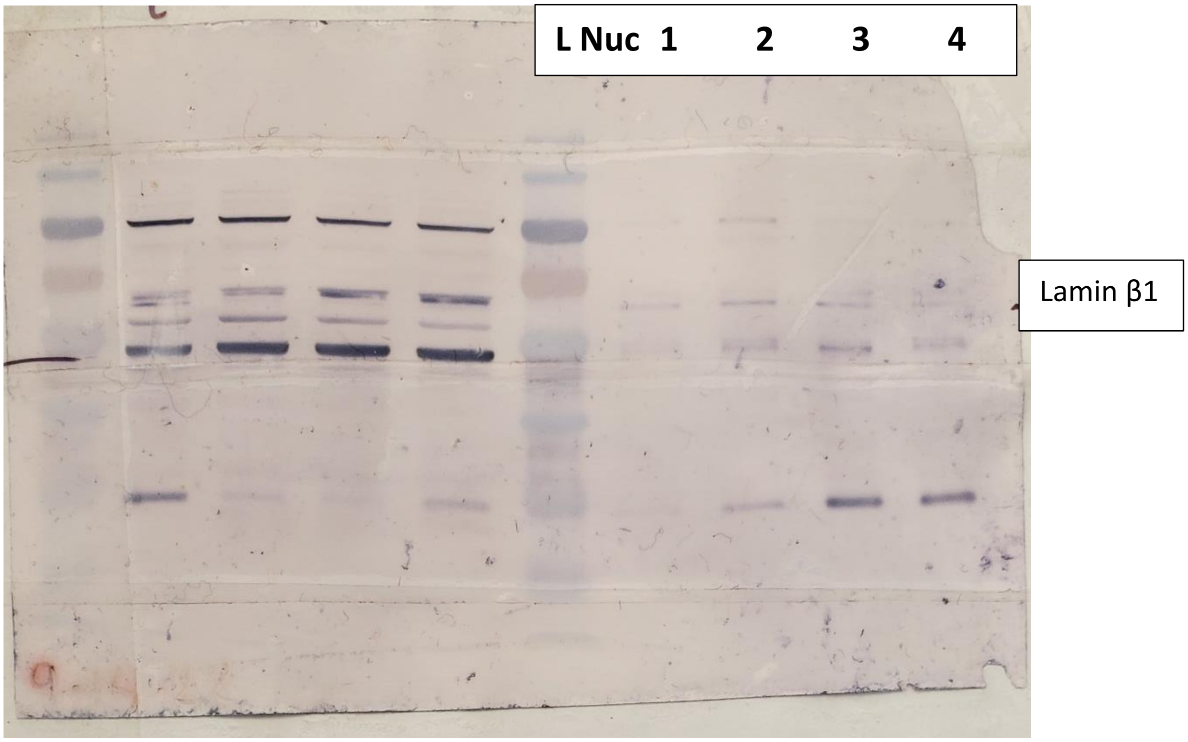
L Nuc 1 2 3 4
Lamin β1

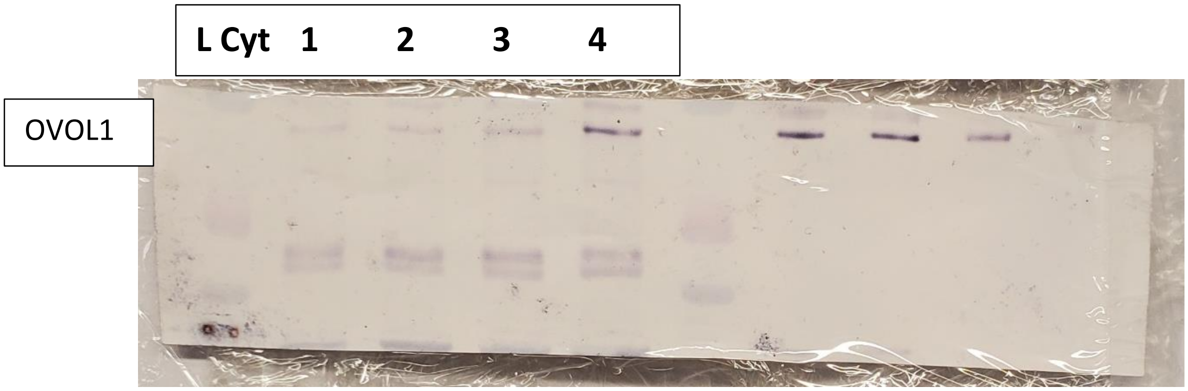
L Cyt 1 2 3 4
OVOL1

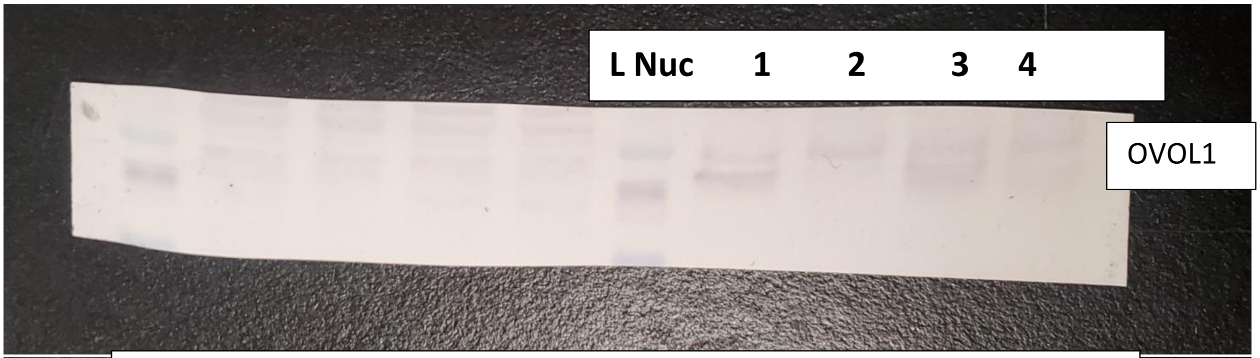
L Nuc 1 2 3 4
OVOL1

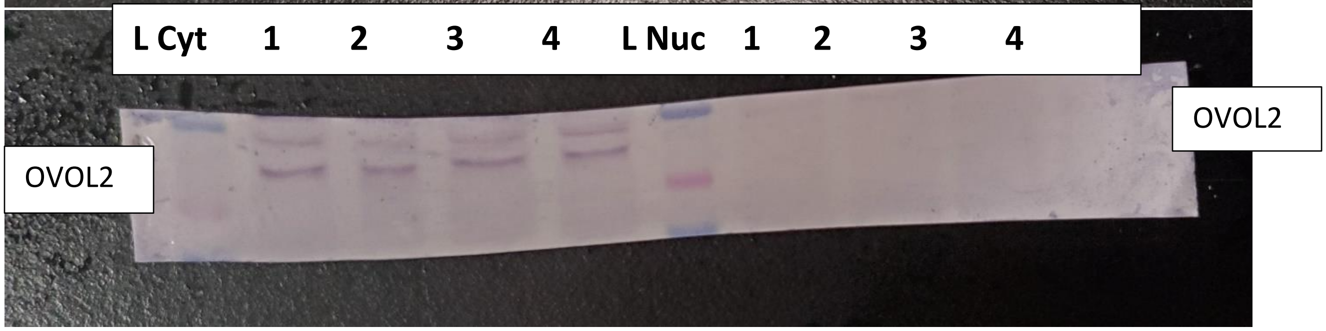
L Cyt 1 2 3 4 L Nuc 1 2 3 4
OVOL2
OVOL2

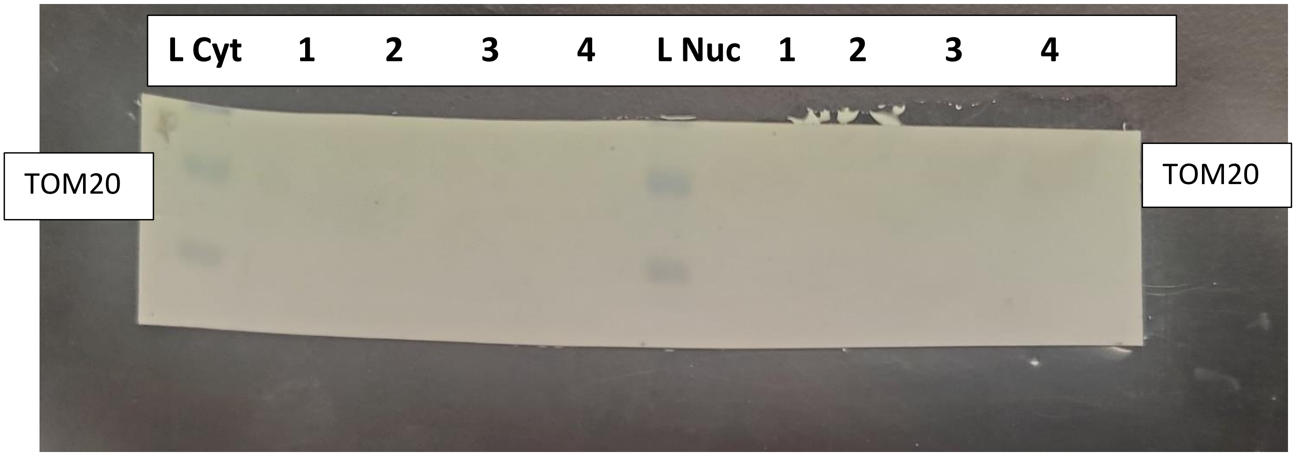
L Cyt 1 2 3 4 L Nuc 1 2 3 4
TOM20
TOM20
